# Historical Mammal Extinction on Christmas Island (Indian Ocean) Correlates with Introduced Infectious Disease

**DOI:** 10.1371/journal.pone.0003602

**Published:** 2008-11-05

**Authors:** Kelly B. Wyatt, Paula F. Campos, M. Thomas P. Gilbert, Sergios-Orestis Kolokotronis, Wayne H. Hynes, Rob DeSalle, Peter Daszak, Ross D. E. MacPhee, Alex D. Greenwood

**Affiliations:** 1 Biological Sciences Department, Old Dominion University, Norfolk, Virginia, United States of America; 2 Department of Biology, University of Copenhagen, Copenhagen, Denmark; 3 Sackler Institute for Comparative Genomics and Division of Invertebrate Zoology, American Museum of Natural History, New York, New York, United States of America; 4 Consortium for Conservation Medicine, Wildlife Trust, New York, New York, United States of America; 5 Vertebrate Zoology, American Museum of Natural History, New York, New York, United States of America; Centre for DNA Fingerprinting and Diagnostics, India

## Abstract

It is now widely accepted that novel infectious disease can be a leading cause of serious population decline and even outright extinction in some invertebrate and vertebrate groups (e.g., amphibians). In the case of mammals, however, there are still no well-corroborated instances of such diseases having caused or significantly contributed to the complete collapse of species. A case in point is the extinction of the endemic Christmas Island rat (*Rattus macleari*): although it has been argued that its disappearance ca. AD 1900 may have been partly or wholly caused by a pathogenic trypanosome carried by fleas hosted on recently-introduced black rats (*Rattus rattus*), no decisive evidence for this scenario has ever been adduced. Using ancient DNA methods on samples from museum specimens of these rodents collected during the extinction window (AD 1888–1908), we were able to resolve unambiguously sequence evidence of murid trypanosomes in both endemic and invasive rats. Importantly, endemic rats collected prior to the introduction of black rats were devoid of trypanosome signal. Hybridization between endemic and black rats was also previously hypothesized, but we found no evidence of this in examined specimens, and conclude that hybridization cannot account for the disappearance of the endemic species. This is the first molecular evidence for a pathogen emerging in a naïve mammal species immediately prior to its final collapse.

## Introduction

Infectious disease is rarely cited as a cause of “complete” (i.e., species-level) extinction in vertebrates, although it is clear that at the population level such diseases (especially ones regarded as emerging within particular taxa) may have far-reaching effects, including outright extirpation [1]. To date, the few well-documented examples of complete extinction in which infectious diseases were demonstrably the main or leading factor mostly concern losses among amphibians [1]; among mammals and birds, extinctions attributable to this cause are poorly corroborated or controversial [2] and, indeed, have been dismissed by some modelers as thoroughly implausible [3]. Progress in understanding will likely come from analyzing cases that can be empirically evaluated in some meaningful way. Unfortunately, most modern-era extinctions that might be considered as potential candidates are hopelessly inadequate for this purpose: either there is no pertinent documentation, or there are no investigable specimens collected before as well as during the time of collapse, or, if there are specimens, there is no available empirical methodology for determining cause of loss. Here we report results of our study of the collapse, allegedly due to introduced infectious disease, of two endemic murines, *Rattus macleari* and *R. nativitatis*, on the isolated landmass of Christmas Island in the eastern Indian Ocean (135 km^2^; 10°29′ S, 105°38′E) almost exactly a century ago.

**Figure 1:**
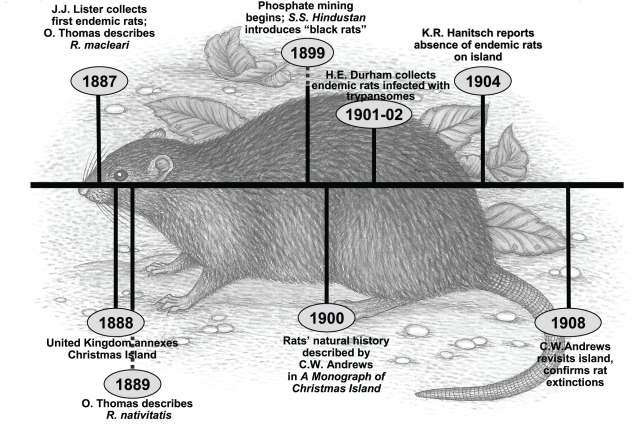
Extinction time line for Christmas Island rats (background drawing of *R. macleari* by Patricia Wynne).

Uninhabited Christmas Island was sighted on several occasions in the two centuries leading up to the first recorded landing in 1857 [4]. However, actual occupation of the island did not occur until the 1890s, following discovery of commercially exploitable deposits of phosphate [4]. The endemic rats of Christmas Island, described as “abundant” when first collected in 1887 [4], [5], but never seen after 1905, are thought to have become completely extinct by 1908 [4]; [6]; Fig. 1]. Discovery to disappearance thus took much less than a quarter-century; indeed, contemporary accounts imply that the actual collapse may have spanned only a few years. Just before their final disappearance, apparently sick individuals of *Rattus macleari* were seen crawling along footpaths and other areas frequented by humans [4]. One explanation proffered at the time by the pioneering tropical parasitologist H.E. Durham [7], [8] was that the animals were suffering from a highly infectious and fatal typanosomiasis, perhaps carried by infected fleas on the black rat (*R. rattus*) thought to have been introduced in 1899 by the *S.S. Hindustan* [4]. According to available evidence [9], the black rat originated in tropical mainland (as opposed to insular) Asia, spreading only much later to Europe and, in recent centuries, to effectively the rest of the world. Durham supported his speculation by gross pathological analysis of a small number of specimens, including ones with pelt characteristics suggestive of hybridization [8]. Because the endemic rats disappeared so quickly, only a small number of specimens were ever collected for scientific study (see [7]); of the few known to still exist, all are housed at just three institutions: the Natural History Museum, London (NHML), and Museum of Zoology of Cambridge University (CMZ), and the Museum of Natural History of Oxford University (OMNH).

The scantiness of the historical records bearing on the demise of the Christmas Island rats raises several questions. One of these is the nature of the organism causing the alleged trypanosomiasis. If it was in fact a trypanosome, what species was involved and how did it prompt the complete extinction of the Christmas Island rats? Another question concerns the status of the animals identified as morphological hybrids. If black rats were hybridizing with the local species, then survival rather than outright extinction would be expected, because trypanosome infections are common and predominantly, but not always [10], nonfatal in the former.

## Results

### Testing for evidence of hybridization between *Rattus macleari* and *R. rattus*

Since the existence of hybridization required a testing procedure independent of that for trypanosomiasis, we first obtained relevant mitochondrial and nuclear DNA sequences from *R. macleari*, *R. rattus* and alleged *R. macleari*×*R. rattus* hybrids in museum collections (N = 18) (*R. macleari* was both more common and more intensively collected than *R. nativitatis* and thus our efforts were focused on this species; see Table 1). Our targets were a single mitochondrial cytochrome b fragment and two fragments of two nuclear DNA genes (designated as RAG1 A and B and GHR A and B) that have been extensively used in phylogenetic analysis of murids [12] (Table 1).

**Table 1:** Information on collected samples, PCR primers and PCR performed in this study.

Sample number	Morphological description^a^	Collection	Collection date	PCR primer name^e^	Primer sequence 5′ to 3′
**E2072**	*Rattus rattus*	Cambridge University	1900–1902	CytB.For2	GATGTGTAGTGTATTGCTA
**E2073**	*Rattus rattus*	Cambridge University	1900–1902	RAG1.A.For	TGCCGCATCTGTGGCAATCA
**E2076**	*Rattus rattus*	Cambridge University	1900–1902	RAG1.A.Rev	TCTTTCGGAAAAGGCTTTGA
**E2078**	*Rattus rattus*	Cambridge University	1900–1902	RAG1.B.For	AGCACCTGTTCTGTAGAATA
**E2079**	*Rattus rattus*	Cambridge University	1900–1902	RAG1.B.Rev	TGCTCAGAAAGGACTTGACC
**E2080**	*Rattus rattus*	Cambridge University	1900–1902	GHR.A.For	CTTCCCTTGGCTCTCTGCAC
**E2074**	*Rattus rattus×Rattus macleari*	Cambridge University	1900–1902	GHR.A.Rev	GCATAAAAGTCAATGTTTGC
**E2075**	*Rattus rattus×Rattus macleari*	Cambridge University	1900–1902	GHR.B.For	AATGTCCGAGACAGCAGATA
**18606**	*Rattus rattus×Rattus macleari*	Oxford U. Mus. Nat. Hist.	1900–1902	GHR.B.For2	CTGAGATGCCTGTCCCAGAC
**18607**	*Rattus rattus×Rattus macleari*	Oxford U. Mus. Nat. Hist.	1900–1902	GHR.B.Rev	AAGCAGTCGCGTTGAGTATA
**18608**	*Rattus rattus×Rattus macleari*	Oxford U. Mus. Nat. Hist.	1900–1902	TRYPA.For	AATTCATTCCGTGCGAAAGC
**18842**	*Rattus rattus×Rattus macleari*	Oxford U. Mus. Nat. Hist.	1900–1902	TRYPA.Rev	GCTGATAGGGCAGTTGTTCG
**E2077**	*Rattus macleari*	Cambridge University	1900–1903	TRYPB.For	ATCAATTTACGTGCATATTC
**18841**	*Rattus macleari*	Oxford U. Mus. Nat. Hist.	1900–1902	TRYPB.Rev	CAGATAACGTGCTGAGGATA
**18843**	*Rattus macleari*	Oxford U. Mus. Nat. Hist.	1900–1902		
**18844**	*Rattus macleari*	Oxford U. Mus. Nat. Hist.	1900–1902		
**18845**	*Rattus macleari*	Oxford U. Mus. Nat. Hist.	1900–1902		
**18846**	*Rattus macleari*	Oxford U. Mus. Nat. Hist.	1900–1902		
**NHM 1899.8.6.28**	*Rattus nativitatis*	Nat. Hist. Mus. London	1897		
**NHM 1899.8.6.29**	*Rattus nativitatis*	Nat. Hist. Mus. London	1897		
**NHM 1888.7.9.5**	*Rattus nativitatis*	Nat. Hist. Mus. London	1888		

Sample number	cytochrome b^f^	PCR Results^b^^,^ c ^,^ d					
		RAG1 A	RAG1 B	GHR A	GHR B	TRYP A	TRYP B^h^
**E2072**		2x/0x	*nd*	*nd*	*nd*		
**E2073**		2x/0x	*nd*	*nd*	*nd*		
**E2076**		2x/0x	*nd*	*nd*	*nd*		
**E2078**		2x/0x	2x/0x	2x/0x	2x/0x		
**E2079**	1x	2x/1x	2x/0x	2x/0x	2x/1x	2x/1x	2x/0x
**E2080**		1x/0x	*nd*	*nd*	*nd*		
**E2074**	1x	2x/0x	2x/0x	3x/0x	2x/0x		1x/0x
**E2075**	1x	2x/2x	2x/0x	2x/0x	3x/0x	1x/0x	
**18606**	1x	2x/0x	2x/0x	4x/0x	2x/0x		
**18607**	1x	1x/1x	2x/0x	2x/0x	4x/0x	2x/1x	2x/1x
**18608**	1x	2x/0x	*nd*	*nd*	*nd*		
**18842**	1x	2x/0x	*nd*	*nd*	*nd*		
**E2077**		1x/2x	*nd*	*nd*	*nd*		1x/0x
**18841**		3x/0x	2x/0x	2x/0x	2x/0x		
**18843**		2x/0x	*nd*	*nd*	*nd*		
**18844**		2x/0x	*nd*	*nd*	*nd*		
**18845**		2x/0x	2x/0x	2x/0x	2x/0x		
**18846**	0x/2x^g^	2x/1x	2x/0x	2x/0x	2x/0x	2x/1x	1x/1x
**NHM 1899.8.6.28**		0x/1x	*nd*	*nd*	*0x/1x*		
**NHM 1899.8.6.29**		0x/1x	*nd*	*nd*	*0x/1x*		
**NHM 1888.7.9.5**		0x/1x	*nd*	*nd*	*0x/1x*		

a Reference [7] of main text.

b number of sequenced PCR reactions at ODU/UC respectively, “nd” entries indicate specific PCR reactions not performed on a given sample.

c Clone sequences available by request to corresponding author.

d Extractions at ODU follow reference 18 and extractions at U of Copenhagen follow reference [14].

e Primers used to generate 377 bp cytochrome b fragment from reference [11].

f The 377 bp cytochrome b fragments were determined from extractions done in Munich, Germany (MediGenomix GmbH).

g The 377 bp amplification did not yield rat sequence with this sample. Cytb.For2 was substituted for the original Forward primer and yielded rat sequences.

h All samples were tested for the presence of trypanosomes. Only those that yielded trypanosome sequences are indicated.

**Figure 2:**
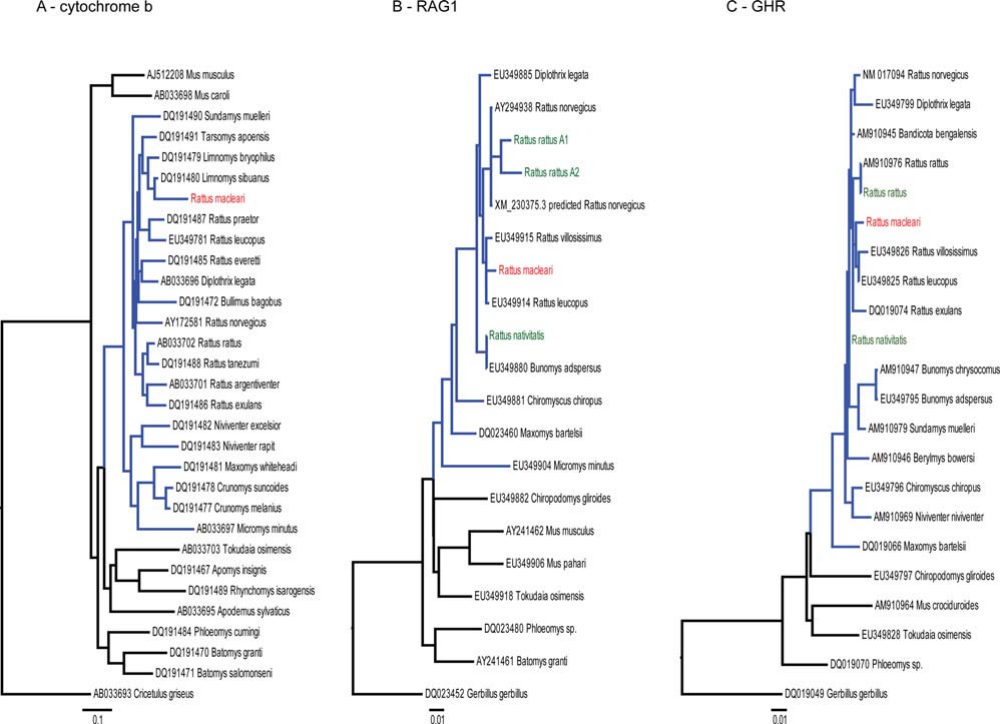
Phylogenetic relationships within tribe Rattini (Muridae: Murinae) based on cytochrome b (A), RAG1 (B), and GHR (C) coding sequences including the nucleotide sequences produced in this study. All trees were estimated in a maximum likelihood framework. Scale bars denote substitutions per site along the branches. Shown in red and green are the rat sequences obtained in this study. The subtree corresponding to the *Rattus* species group *sensu lato* is colored in blue for clarity.

Sequences were obtainable from all 18 rat samples (100% success rate) with the RAG1 A primers. On the basis of fixed differences in recovered cytochrome b and RAG1 sequences (Table 2), we determined that the samples could be exhaustively divided into two groups. More precisely, modern *Rattus rattus* and the alleged hybrids were found to differ in a distinct and consistent manner from specimens designated as *R. macleari* on museum labels, with little or no within-group variation (0-2 difference per fragment) (Table 2). Given the lack of within-group differences for RAG 1A, genes RAG 1 B, GHR A and B were sampled in a subset of specimens, with identical results, as determined by comparing recovered sequences to those for *R. rattus* in GenBank and by performing relevant phylogenetic analysis (Fig. 2). Results with the different genes gave a consistent result indicating *R. macleari* was indeed distinct from *R. rattus*. We conclude that the absence of consistent genetic differences between *R. rattus* and the putative hybrids indicates that the latter are simply morphological variants of the former, which is consistent with the observation that *R. rattus* is a notably polymorphic species [13]. If intensive hybridization had actually occurred, it would have had to happen within a very short period, as the endemic rats became extinct within a maximum of 9 years subsequent to black rat introduction. In any case, it would be expected that at least some individuals—and in particular the morphological hybrids—would harbor alleles from both species: no evidence of this can be seen in the genetic information available.

**Table 2:** Identified polymorphisms in Christmas Island rats for Cytb, RAG1 and GHR.

Species designation	Sample number	Cytb^a^									RAG1											GHR			
											A^c^						B					A		B	
		13	20	22	32	34	40	49	50	52	1	13	14	40	41	52	10	13	37	46	76	60	108	35	50
***Rattus rattus***	E2079^b^	T	G	A	A	T	T	C	T	G	C	C	A	C	G	C		G	C	G	G	A	G	T	C
	E2072, E2080	nd	nd	nd	nd	nd	nd	nd	nd	nd	.	.	.	.	.	.	nd	nd	nd	nd	nd	nd	nd	nd	nd
	E2073	nd	nd	nd	nd	nd	nd	nd	nd	nd	.	.	.	.	.	.	nd	nd	nd	nd	nd	nd	nd	nd	nd
	E2076	nd	nd	nd	nd	nd	nd	nd	nd	nd	.	.	G	.	.	.	nd	nd	nd	nd	nd	nd	nd	nd	nd
	E2078^d^	nd	nd	nd	nd	nd	nd	nd	nd	nd	.	.	A/G	.	.	.		.	.	.	.	.	.	.	.
**Putative hybrids**	E2074,18606	.	.	.	.	.	.	.	.	.	.	.	.	.	.	.		.	.	.	.	.	.	.	.
	E2075^d^	.	.	.	.	.	.	.	.	.			A/G	.	.	.		.	.	.	.	.	.	.	.
	18607	.	.	.	G	.	.	A	.	A	.	.	G	.	.	.		A	.	.	A	.	.	.	.
	18608, 18842	.	.	.	G	.	.	A	.	A	.	.	.	.	.	.	nd	nd	nd	nd	nd	nd	nd	nd	nd
***Rattus macleari***	E2077, 18843, 18844	nd	nd	nd	nd	nd	nd	nd	nd	nd	.	T*	G	.	.	T*	nd	nd	nd	nd	nd	nd	nd	nd	nd
	18841, 18845	nd	nd	nd	nd	nd	nd	nd	nd	nd	.	T*	G	.	.	T*		.	T*	T*	.	G*	A*	C*	T*
	18846	C*	A*	G*	.	C*	C*	A	C*	.	.	T*	G	.	.	T*	T*	.	T*	T*	.	G*	A*	C*	T*
***Rattus nativitatis***	1899.8.6.28, 1899.8.6.29	nd	nd	nd	nd	nd	nd	nd	nd	nd	T*	.	G	T*	.	.	nd	nd	nd	nd	nd	nd	nd	C*	.
	1888.7.9.5^d^	nd	nd	nd	nd	nd	nd	nd	nd	nd	T*	.	G	T*	A/G	.	nd	nd	nd	nd	nd	nd	nd	C*	.

a Number is from the first base after the primer of each PCR product. Sequences not obtained are marked “nd”. Dots represent identity to the reference sequence.

b E2079 was used as the reference sequence. Differences between *R. macleari*, *R. nativitatis* and *R. rattus*/putative hybrids are marked “^*^”.

c A and B refer to PCR products for RAG1 and GHR (see Table 1).

d A/G indicates potential alleles for the individual loci i.e. approximately half the clones had one or the other base.

### Evidence of trypanosome infection in invasive black rats and endemic rats

**Figure 3:**
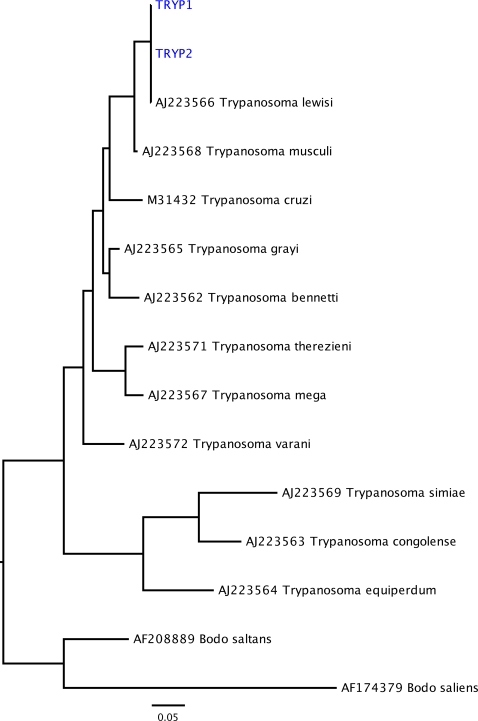
Phylogenetic relationships among trypanosome sequences bases on 18S rDNA sequences. Scale bars denote substitutions per site along branches. Blue-colored sequences are the trypanosome sequences obtained in this study.

Two primer pairs (TrypA and TrypB) targeting the kinetoplastid 18S rDNA region (Table 1) were used to investigate whether trypanosomal DNA was present in any of the specimens. All 21 samples were tested, including three examples of *R. nativitatis*, which were collected prior to the introduction of black rats to Christmas Island. Although it was not expected that all specimens would return a positive signal for trypanosomes, since even highly infectious pathogens rarely exhibit 100% successful infection rates, we did expect OMNH 18846 to test positive because this was one of the animals Durham reported as displaying firm evidence of trypanosome infection [7]. In the event, six of the rats, including OMNH 18846, yielded trypanosome sequences. Five displayed unambiguous (100%) matches to published sequences for *Trypanosoma lewisi*, a known murine-infecting trypanosome; the remaining sample displayed a 3 bp deletion in the fragment amplified and thus could not be unambiguously characterized (Table 3). Unsurprisingly, as there were no differences between the GenBank sequence and those recovered from Christmas Island rats (except for the one instance of a 3 bp deletion), phylogenetic analysis unequivocally grouped them within *T. lewisi* (FIG. 3). Several of the infected rats were independently retested in two separate laboratories: for three samples our results were fully validated, but for three others validation must be regarded as tentative because only one (rather than both) laboratories reported a single replicate positive result—an effective illustration of the difficulties in working with less than single-copy pathogenic DNA from archival samples [14] (Table 1). Although a free-living kinetoplastid, *Bodo saliens*, was detected among the clones, this environmental contaminant could be easily distinguished from obligate parasitic trypanosomes at the sequence level. All morphologically defined subgroups (*R. rattus*, alleged hybrid, and *R. macleari*) contained *T. lewisi* DNA, confirming all three were susceptible to the infection.

**Table 3:** Trypanosome sequences obtained from the Christmas Island rats.

Species designation	Sample number	A								B		
		1^a^	4	58	9–15	18	21–23	26	29	1–5	7–35	38–42
***Trypanosoma lewisi***	AJ223566	C	A	TTCT	- - - - -TT	G	- - -	C	T	TTTTT	G -14 bp del- TCCTCGCAAGAGGT	TTTTA
***Trypanosoma musculi***	AJ223568	.	.	. . . .	TT - - - - -	.	- - -	.	.	. . . . . .	GTCCTC GCA - AGAGGT	- - - - -
***Trypanosoma cruzi***	M31432	.	G	TTTT	TT - - - A -	.	TAG	.	.	. . . . . .	TCTTCGTTTTTTTT ACGGCGAGGA	TTTAA
***Bodo saliens***	AF174379	.	T	TC- -	- - - - - A A	.	- - -	.	C	CGTTG	CTTT ACCG	AATAT
***Bodo saltans***	AF208889	T	T	CT- -	- - - - - - A	A	- - -	A	.	TACTG	CCTTC GGG	GTCAA
***Rattus rattus***	E2079	.	.	. . . .	. . . . . . .	.	. . .	.	.	. . . . .	. . . . . . . . . . . . . . . . . . . . . . . . . . . . .	. . . . .
**Putative hybrids**	18607	.	.	. . . .	. . . . . . .	.	. . .	.	.	. . . . .	. . . . . . . . . . . . . . . . . . . . . . . . . . . . .	. . . . .
	E2074^d^	nd	nd	nd	nd	nd	nd	nd	nd	. . . . .	. . . . . . . . . . . . . . . . . . . . . . . . . . . . .	. . . . .
	E2075^d^	.	.	- - -T	. . . . . . .	.	. . .	.	.	nd	nd	nd
***Rattus macleari***	E2077^d^	nd	nd	nd	nd	nd	nd	nd	nd	. . . . .	. . . . . . . . . . . . . . . . . . . . . . . . . . . . .	. . . . .
	18846	.	.	. . . .	. . . . . . .	.	. . .	.	.	. . . . .	. . . . . . . . . . . . . . . . . . . . . . . . . . . . .	. . . . .

a Numbering begins from the first base after the 5′ PCR primer. *T. lewisi* is used as a reference sequence. Identities are shown as dots and differences as the differing bases except for long stretches of insertions or deletions where the entire stretch of sequences are shown. Long deletions are given as numbers of deleted bases.

b A and B refer to the individual PCR products amplified with the two independent PCR primers (see Table 1).

c Sequences not determined are shown as “nd”.

d The results for these samples were not replicated.

If, as alleged by Durham [7], fleas from ship-borne black rats introduced in 1899 were the transmission vector, endemic rat samples collected prior to 1899 should be free of trypanosome infection. Three such specimens were examined (all *R. nativitatis*, collected in 1888; no pre-contact *R. macleari* samples are available for study). Even after 60 cycles of PCR no trypanosome sequences could be detected in the pre-black rat introduction samples; by contrast, nuclear DNA was amplifiable, indicating that DNA was present in all three samples (Table 2 and Fig. 2).

## Discussion

We did not test for the species-level distinctiveness of *Rattus macleari* vs. *R. nativitatis*; attribution of specimens to one or the other taxon was based on original museum labels. However, as reported above we did test for the distinctiveness of the island endemics as compared to *R. rattus* and the formerly ambiguous grouping of “hybrids”, all of which tested as true *R. rattus*. Although sampling limitations were admittedly severe, in light of the consistency of our results the notion that hybridization between *R. rattus* and *R. macleari* resulted in the disappearance of phenotypically pure *R. macleari* can be considered unlikely.

Black rats are often implicated in arguments concerning competitive exclusion and extinction on islands: they are notably omnivorous, and will feast on practically anything, including insects, bird eggs, bird fledglings, small lizards, land snails, mollusks, land crabs and even turtle hatchlings [15]. In the central Pacific there is evidence that introduced black rats (and Norwegian rats as well) have spurred extirpations and even extinctions among sedentary oceanic birds, especially rails [15]. In light of this it may be wondered whether the Christmas Island endemics might have been added to the diet of black rats, once the latter managed to get ashore, and that predation, rather than introduced disease, could have been the actual coup de grâce looming behind the extinction of *Rattus macleari* and *R. nativitatis*. Although there is of course no evidence directly bearing on this question, it is of interest that in other island settings where black rats share habitat with other murid species, extinction of their confamilials has not necessarily occurred. Thus according to Spennemann's comprehensive data [15], on each of the eight islands on which black rats occur within the Marshall Islands group, there is also a population of the Pacific rat *Rattus exulans*. In some instances, co-existence must have extended over centuries, indicating that these populations have reached accommodation. In short, mere presence of invasive black rats does not invariably lead to extinction of other small vertebrates, and there is no *a priori* reason to believe that this is what happened on Christmas Island. Indeed, the only other endemic mammal on the island at the time of British occupation, the Christmas Island shrew (*Crocidura trichura*), although quite rare (or rarely encountered), was still extant as of 1985 [16]. Given the host specificity of trypanosomes, it would not be expected that shrews would be susceptible to rat trypanosomes and thus competition or predation would be the likelier scenario for this group. Yet, they persisted while the trypanosome-susceptible species did not.

The presence of detectable murid trypanosome sequence in *R. rattus* and *R. macleari* samples indicates that the parasite was present in both populations. By contrast, *R. nativitatis* samples collected before the introduction of black rats did not yield trypanosome sequences. While this absence of evidence cannot be considered decisive given the few samples available for analysis, it is plausible that long-isolated endemic rat species would have been immunologically naïve and therefore highly susceptible to common diseases carried by ectoparasites of other murines. Modern evidence shows that most *R. rattus* infected with *T. lewisi* will survive exposure, but there is nevertheless a mortality rate associated with infection [10]: depending on the time of infection, in pregnant rats *T. lewisi* can cause death or termination of pregnancy. It is also acknowledged that, when trypanosomes cross species boundaries in mammals, they may cause evident morbidity [17].

In summary, the DNA evidence presented in this paper is consistent with the following conclusions: (1) *R. macleari* was a species distinct from the black rat, and that (in the absence of detectable hybrids or exotic alleles) the murines of Christmas Island must have long existed in isolation from black rats and their diseases; (2) the introduction of a candidate pathogen, *Trypanosoma lewisi*, to immunologically naïve murine hosts on the island around 1900 is consistent with contemporary reports of widespread morbidity and perhaps also extensive mortality that so reduced endemic populations that they collapsed to the point of complete extinction within the space of not more than 9 years. This study represents, for mammals, the first verified correlation in time of novel pathogen introduction and species-level extinction.

## Materials and Methods

### Samples

Approximately 1 square inch of skin was taken from each archival rat specimen using scissors which were sterilized between each rat sampling. Efforts were made in every case to obtain samples displaying blood vessels in order to maximize the chance of detecting blood-borne pathogens. Details of samples collected are shown in Table 1. Masks and gloves were used throughout and efforts were made to avoid any cross contamination of samples during sampling, such as sterilizing instruments between each sample and changing gloves frequently.

### DNA extraction, PCR and sequencing

Extractions in Norfolk were carried out in a room dedicated to ancient DNA work in a CleanSpot PCR hood (Coy Laboratory, MI) following a protocol similar to that in [18]. Approximately 0.5 gram of skin was used per extraction. The room had never been previously used for molecular biological work. Separating rooms used for processing ancient DNA samples and performing modern molecular biological investigations is a useful way of minimizing contamination risk [19]. Amplified PCR products never entered the clean room nor did modern DNA. Extraction of DNA from the skin samples was done using GeneClean Ancient DNA Kits (MP Biomedical, CA) according to manufacturer's instructions. Mock extractions were performed to control for contamination introduced during extraction.

For most samples, multiple independent PCRs were performed (See Table 1 for the number of PCRs performed per PCR and results replicated per independent laboratory). Most PCRs were performed at least twice per PCR primer set. PCR amplification was performed for 40 cycles using HiFi Supermix (Invitrogen) which is a Taq mix that is known to perform well on ancient DNA extracts [20]. Annealing temperatures were chosen based on the Tm of the primers. All PCR products were cloned into T overhang vectors, transformed into competent bacteria, with positives colonies identified by colony PCR and multiple clones per PCR product sequenced. Direct sequencing can lead to an erroneous sequence due to contamination and DNA damage in the extract. Cloning and sub-sampling individual representative amplified sequences provides a better representation of the original template amplified [21]; none of the consensus sequences generated in this study were determined from direct sequencing. Primers sequences are shown in Table 1 and clone sequences in Supplemental File S1 in FASTA format.

To independently reproduce a portion of the data, a subset of samples was sent to the University of Copenhagen. Extractions were performed at the University of Copenhagen in a dedicated ancient DNA laboratory using the Qiagen DNEasy extraction kit (Qiagen, Valencia, CA). All PCR products were purified using the Qiagen Qiaquick PCR clean up kit, then cloned using the Topo TA cloning system (Invitrogen, Carlsbad, CA). Following colony PCR, inserts of approximately the correct size were sequenced using vector primers M13F/M13R produced by the commercial Macrogen facility (Macrogen, Seoul, Korea). Sequences have been deposited in GenBank under accession nos. EU814873–EU814886.

### Sequence analysis

Nucleotide sequences were aligned in MAFFT 6 using the E-NS-i method [22] with homologs from GenBank (see phylogenetic tree figures for accession numbers), and then inspected and edited, where necessary, in Se-Al 2.0a11 (available from http://tree.bio.ed.ac.uk/software/seal). Alignments are provided as FASTA files in Supplemental File S2, S3, S4 and S5 for Cytb, RAG1, GHR and trypanosome sequences respectively. With respect to the mitochondrial cytochrome b gene, we increased taxonomic representation (30 taxa+*R. macleari*) in order to minimize the error in phylogenetic positioning of taxa due to the short sequence length of our ancient sequences, as well as the substitution saturation present in this mitochondrial alignment [23]; *Cricetulus griseus* (Cricetidae) was used as outgroup. RAG1 sequences originating from a recent study [24] of 16 taxa were downloaded from GenBank. GHR exon sequences were retrieved for 20 taxa from GenBank corresponding to two recent studies [24], [25]. *Gerbillus gerbillus* (Muridae: Gerbillinae) was used as outgroup in the nuclear datasets. The phylogenetic position of our sequences was examined in a maximum likelihood (ML) framework for all loci in RAxML 7.0.4 [26]. In the latter application, initial trees are created by random stepwise taxon addition and built using maximum parsimony (MP). Tree length is optimized through two subtree pruning-regrafting moves and these MP trees are used as starting trees for the ML search. We used the general time-reversible substitution model [27], [28] along with Γ-distributed rate heterogeneity [29], as implemented in RAxML.

## Supporting Information

Supplemental File S1 (0.18 MB DOC)

Supplemental File S2 (0.04 MB TXT)

Supplemental File S3 (0.02 MB TXT)

Supplemental File S4 (0.02 MB TXT)

Supplemental File S5 (0.04 MB TXT)
