# Correction: Historical Mammal Extinction on Christmas Island (Indian Ocean) Correlates with Introduced Infectious Disease

**DOI:** 10.1371/annotation/9fbe9687-682e-4010-97e4-139b33343d34

**Published:** 2009-01-05

**Authors:** Kelly B. Wyatt, Paula F. Campos, M. Thomas P. Gilbert, Sergios-Orestis Kolokotronis, Wayne H. Hynes, Rob DeSalle, Stanley J. Ball, Peter Daszak, Ross D. E. MacPhee, Alex D. Greenwood

Dr. Stanley J. Ball was not included in the author byline. He should be listed as the seventh author and affiliated with School of Life Sciences, Kingston University, Kingston upon Thames, Surrey, United Kingdom. The contributions of this author are as follows: Contributed reagents/materials/analysis tools. 

