# Supplementary material for: Historical Mammal Extinction on Christmas Island (Indian Ocean) Correlates with Introduced Infectious Disease
Source: PLoS One. 2008 Nov 5;3(11):e3602. doi: 10.1371/journal.pone.0003602 (PMC2572834; doi:10.1371/journal.pone.0003602)
Supplement: Supplemental File S1 — (0.18 MB DOC) [file pone.0003602.s001.doc]

**Supplemental Clone Sequence Database Legend:**

Each sequence derived from individual cloned PCR products from each Christmas Island specimen for cytochrome b, RAG1, GHR and trypanosome 18S rDNA are contained within this FASTA file. Specimen names are followed by the letter A-D indicating the individual PCR amplification from which the clones are derived. Thus, those fragments with clones A-D were amplified 4 times and clones sequenced from each product. PCR’s performed and sequenced at the University of Copenhagen (Denmark) are indicated. Cytochrome b clones are labeled “f “ and “r” indicating that each clone was sequenced in both directions.

**Supplemental Clone Sequence Database:**

**>Cytochrome b**

**>18846.A.1.Denmark**

**TTTTGGCTCTCTCCTAGGAATGTGCCTTATAATCCAAATCATCACAGGACTGTTTC----**

**------------------------------------------------------------**

**--------------------------------**

**>18846.A.2.Denmark**

**TTTTGGCTCTCTCCTAGGAATGTGCCTTATAATCCAAATCATCACAGGACTGTTTC----**

**------------------------------------------------------------**

**-------------------------------**

**>18846.A.3.Denmark**

**TTTTGGCTCTCTCCTAGGAATGTGCCTTATAATCCAAATCATCACAGGACTGTTTC----**

**------------------------------------------------------------**

**-------------------------------**

**>18846.A.4.Denmark**

**TTTTGGCTCTCTCCTAGGAATGTGCCTTATAATCCAAATCATCACAGGACTGTTTC----**

**------------------------------------------------------------**

**-------------------------------**

**>18846.A.5.Denmark**

**TTTTGGCTCTCTCCTAGGAATGTGCCTTATAATCCAAATCATCACAGGACTGTTTC----**

**------------------------------------------------------------**

**-------------------------------**

**>18846.A.6.Denmark**

**TTTTGGCTCTCTCCTAGGAATGTGCCTTATAATCCAAATCATCACAGGACTGTTTC----**

**------------------------------------------------------------**

**-------------------------------**

**>18846.A.7.Denmark**

**TTTTGGCTCTCTCCTAGGAATGTGCCTTATAATCCAAATCATCACAGGACTGTTTC----**

**------------------------------------------------------------**

**--------------------------------**

**>18846.A.8.Denmark**

**TTTTGGCTCTCTCCTAGGAATGTGCCTTATAATCCAAATCATCACAGGACTGTTTC----**

**------------------------------------------------------------**

**-------------------------------**

**>18846.A.9.Denmark**

**TTTTGGCTCTCTCCTAGGAATGTGCCTTATAATCCAAATCATCACAGGACTGTTTC----**

**------------------------------------------------------------**

**-------------------------------**

**>18846.A.10.Denmark**

**TTTTGGCTCTCTTCTAGGAGTATGCCTTATAATCCAAATCATCACAGGACTGTTTC----**

**------------------------------------------------------------**

**-------------------------------**

**>18846.A.11.Denmark**

**TTTTGGCTCTCTTCTAGGAGTATGCCTTATAATTCAAATTATCACAGGTTTGTTTC----**

**------------------------------------------------------------**

**-------------------------------**

**>18846.A.12.Denmark**

**TTTTGGCTCTCTTCTAGGAGTATGCCTTATAATTCAAATTATCACAGGCTTGTTTC----**

**------------------------------------------------------------**

**-------------------------------**

**>18846.A.13.Denmark**

**TTTTGGCTCTCTTCTAGGAGTATGCCTTATAATTCAAATTATCACAGGCTTGTTTC----**

**------------------------------------------------------------**

**-------------------------------**

**>18846.A.14.Denmark**

**TTTTGGCTCTCTTCTAGGAGTATGCCTTATAATTCAAATTATCACAGGCTTGTTTC----**

**------------------------------------------------------------**

**-------------------------------**

**>18846.B.1.Denmark**

**TTTTGGCTCTCTCCTAGGAATGTGCCTTATAATCCAAATCATCACAGGACTGTTTC----**

**------------------------------------------------------------**

**-------------------------------**

**>18846.B.2.Denmark**

**TTTTGGCTCTCTCCTAGGAATGTGCCTTATAATCCAAATCATCACAGGACTGTTTC----**

**------------------------------------------------------------**

**-------------------------------**

**>18846.B.3.Denmark**

**TTTTGGCTCTCTCCTAGGAATGTGCCTTATAATCCAAATCATCACAGGACTGTTTC----**

**------------------------------------------------------------**

**-------------------------------**

**>18846.B.4.Denmark**

**TTTTGGCTCTCTCCTAGGAATGTGCCTTATAATCCAAATCATCACAGGACTGTTTC----**

**------------------------------------------------------------**

**-------------------------------**

**>18846.B.5.Denmark**

**TTTTGGCTCTCTCCTAGGAATGTGCCTTATAATCCAAATCATCACAGGACTGTTTC----**

**------------------------------------------------------------**

**-------------------------------**

**>18846.B.6.Denmark**

**TTTTGGCTCTCTCCTAGGAATGTGCCTTATAATCCAAATCATCACAGGACTGTTTC----**

**------------------------------------------------------------**

**-------------------------------**

**>18846.B.7.Denmark**

**TTTTGGCTCTCTTCTAGGAGTATGCCTTATAGTTCAAATTATCACAGGATTATTTC----**

**------------------------------------------------------------**

**-------------------------------**

**>18846.B.8.Denmark**

**TTTTGGCTCTCTTCTAGGAGTATGCCTTATAATTCAAATTATCACAGGCTTGTTTC----**

**------------------------------------------------------------**

**-------------------------------**

**>**

**>E2079.2f**

**TTTTGGCTCTCTTCTAGGAGTATGCCTTATAATTCAAATTATCACAGGCTTGTTTCTAGC**

**AATACACTACACATCCGACACTTTAACAGCATTCTCNTNNNTTACTCACATCTGCCGAGA**

**CGTAAACTACGGCTGACTAATNNTNTACTTACATGCCAACGGAGCCTCAATATTCTTTAT**

**CTGCTTATTCCTCCATGTAGGCCGAGGGATATACTACGGATCCTACACCTTCTTAGAAAC**

**ATGAAACATTGGAATTATCCTACTATTTGCAGTCATAGCAACCGCATTCATAGGTTATGT**

**ACTCCCA**

**>E2079.2r**

**TTTTGGCTCTCTTCTAGGAGTATGCCTTATAATTCAAATTATCACAGGCTTGTTTCTAGC**

**AATACACTACACATCCGACACTTTAACAGCATTCTCATCAGTTACTCACATCTGCCGAGA**

**CGTAAACTACGGCTGACTAATCCGATACTTACATGCCNACGGAGCCTCAATATTCTTTAT**

**CTGCTTATTCCTCCATGTAGGCCGAGGGATATACTACGGATCCTACACCTTCTTAGAAAC**

**ATGAAACATTGGAATTATCCTACTATTTGCAGTCATAGCAACCGCATTCATAGGTTATGT**

**ACTCCCA**

**>E2079.3f**

**CTTCGGCTCACTCCTTGGCGCCTGCCTGATCCTCCAAATCACCACAGGACTATTCCTAGC**

**CATGCACTACTCACCAGACGCCTCAACCGCCTTTTCNTNANTCGCCCACATCACTCGAGA**

**CGTAAATTATGGCTGAATCATCCGCTACCTTCACGCCAATGGCGCCTCAATATTCTTTAT**

**CTGCCTCTTCCTACACATCGGGCGAGGCCTATATTACGGATCATTTCTCTACTCAGAAAC**

**CTGAAACATCGGCATTATCCTCCTGCTTGCAACTATAGCAACAGCCTTCATAGGCTATGT**

**CCTCCCG**

**>E2079.3r**

**CTTCGGCTCACTCCTTGGCGCCTGCCTGATCCTCCAAATCACCACAGGACTATTCCTAGC**

**CATGCACTACTCACCAGACGCCTCAACCGCCTTTTCATCAATCGCCCACATCACTCGAGA**

**CGTAAATTATGGCTGAATCATCCGCTACCTTCACGCCAATGGCGCCTCAATATTCTTTAT**

**CTGCCTCTTCCTACACATCGGGCGAGGCCTATATTACGGATCATTTCTCTACTCAGAAAC**

**CTGAAACATCGGCATTATCCTCCTGCTTGCAACTATAGCAACAGCCTTCATAGGCTATGT**

**CCTCCCG**

**>E2079.5f**

**CTTCGGCTCACTCCTTGGCGCCTGCCTGATCCTCCAAATCACCACAGGACTATTCCTAGC**

**CATGCACTACTCACCAGACGCCTCAACCGCCTTTTCATCAATCGCCCACATCACTCGAGA**

**CGTAAATTATGGCTGAATCATCCGCTACCTTCACGCCAAANGNGCCTCAATATTCTTTAT**

**CTGCCTCTTCCTACACATCGGGCGAGGCNTATATTACGGATCATTTCTCTACTCAGAAAC**

**CTGAAACATCGGCATTATCCTCCTGCTTGCAACTATAGCAACAGCCTTCATAGGCTATGT**

**CCTCCCG**

**>E2079.5r**

**CTTCGGCTCACTCCTTGGCGCCTGCCTGATCCTCCAAATCACCACAGGACTATTCCTAGC**

**C-TGCACTACTCACCAGACGCCTCAACCGCCTTTTCATCAATCGCCCACATCACTCGAGA**

**CGTANATTATGGCTGAATCATCCGCTACCTTCACGCCAATGGCGCCTCAATATTCTTTAT**

**CTGCCTCTTCCTACACATCGGGCGAGGCCTATATTACGGATCATTTCTCTACTCAGAAAC**

**CTGAAACATCGGCATTATCCTCCTGCTTGCAACTATAGCAACAGCCTTCATAGGCTATGT**

**CCTCCCG**

**>E2079.7f**

**TTTTGGCTCTCTTCTAGGAGTATGCCTTATAATTCAAATTATCACAGGCTTGTTTCTAGC**

**AATACACTACACATCCGACACTTTAACAGCATTCTCATCATTTACTCACATCTGCCGAGA**

**CGTAAACTACGGCTGACTAATCCGATACTTACATGCCAACGGAGCCTCAATATTCTTTAT**

**CTGCTTATTCCTCCATGTAGGCCGAGGGATATACTACGGATCCTACACCTTCTTAGAAAC**

**ATGAAACATTGGAATTATCCTACTATTTGCAGTCATAGCAACCGCATTCATAGGTTATGT**

**ACTCCCA**

**>E2079.7r**

**TTTTGGCTCTCTTCTAGGAGTATGCCTTATAATTCAAATTATCACAGGCTTGTTTCTAGC**

**AATACACTACACATCCGACACTTTAACAGCATTCTCATCAGTTACTCACATCTGCCGAGA**

**CGTAAACTACGGCTGACTAATCCGATACTTACATGCCAACGGAGCCTCAATATTCTTTAT**

**CTGNTTATTCCTCCATGTAGGCCGAGGGATATACTACGGATCCTACACCTTCTTAGAAAC**

**ATGAAACATTGGAATTATCCTACTATTTGCAGTCATAGCAACCGCATTCATAGGTTATGT**

**ACTCCCA**

**>E2079.8f**

**TTTTGGCTCTCTTCTAGGAGTATGCCTTATAATTCAAATTATCACAGGCTTGTNTCTAGC**

**AATACACTACACATCCGACACTTTAACAGCATTCTCATCANTTACTCACATCTGCCGAGA**

**CGTAAACTACGGCTGACTAATCCNATACTTACATGCCAACGGAGCCTCAATATTCTTTAT**

**CTGCTTATTCCTCCATGTAGGCCGAGGGATATACTACGGATCCTACACCTTCTTAGAAAC**

**ATGAAACATTGGAATTATCCTACTATTTGCAGTCATAGCAACCGCATTCATAGGTTATGT**

**ACTCCCA**

**>E2079.8r**

**TTTTGGCTCTCTTCTAGGAGTANGCCTTATAATTCAAATTATCACAGGCTTGTTTCTAGC**

**AATACACTACACATCCGACACTTTAACAGCATTCTCATCAGTTACTCACATCTGCCGAGA**

**CGTAAACTACGGCTGACTAATCCGATACTTACATGCCAACGGAGCCTCAATATTCTTTAT**

**CTGCTTATTCCTCCATGTAGGCCGAGGGATATACTACGGATCCTACACCTTCTTAGANAC**

**ATGAAACATTGGAATTATCCTACTATTTGCAGTCATAGCAACCGCATTCATAGGTTATGT**

**ACTCCCA**

**>**

**>E2074.7f**

**CTTCGGCTCACTCCTTGTCGCCTGCCTGATCCTCCAAATCACCACAGGACTATTCCTAGC**

**CATGCACTACTCACCAGACGCCTCAACCGCCTTTTCATCAATCGCCCACATCACTCGAGA**

**CGTAAATTATGGCTGAATCATCCGCTACCTTCACGCCAATGGCGCCTCAATATTCTTTAT**

**CTGCCTCTTCCTACACATCGGGNGAGGCCTATANTACGGATCATTTCTCTACTCAGAAAC**

**CTGAAACATCGGCATTATCCTCCTGCTTGCAACTATAGCAACAGCCTTCATAGGCTATGT**

**CCTCCCG**

**>E2074.7r**

**CTTCGGCTCACTCCTTGTCGCCTGCCTGATCCTCCAAATCACCACAGGACTATTCCTAGC**

**CATGCACTACTCACCAGACGCCTCAACCGCCTTTTCATCAATCGCCCACATCACTCGAGA**

**CGTAAATTATGGCTGAATCATCCGCTACCTTCACGCCAATGGCGCCTCAATATTCTTTAT**

**CTGCCTCTTCCTACACATCGGGCGAGGCCTATATTACGGATCATTTCTCTACTCAGAAAC**

**CTGAAACATCGGCATTATCCTCCTGCTTGCAACTATAGCAACAGCCTTCATAGGCTATGT**

**CCTCCCG**

**>E2074.8f**

**TTTTGGCTCTCTTCTAGGAGTACGCCTTATAATTCAAATTATCACAGGCTTGTTTCTAGC**

**AATACACTACATATCCGACACTTTAACAGCATTCTCATCAGTTACTCACATCTGCCGAGA**

**CGTAAACTACGGCTGACTAATCCGATACTTACATGCCAACGGAGCCTCAATATTCTTTAT**

**CTGCTTATTCCTCCATGTAGGCNGANGNATATACTACGGATCCTACACCTTCTTAGAAAC**

**ATGAAACATTGGAATTATCCTACTATTTGCAGTCATAGCAACCGCATTCATAGGTTATGT**

**ACTCCCA**

**>E2074.8r**

**TTTTGGCTCTCTTCTAGGAGTACGCCTTATAATTCAAATTATCACAGGCTTGTNTCTAGC**

**AATACACTACATATCCGACACTTTAACAGCATTCTCATCAGTTACTCACATCTGCCGAGA**

**CGTAAACTACGGCTGACTAATCCGATACTTACATGCCAACGGAGCCTCAATATTCTTTAT**

**CTGCTTATTCCTCCATGTAGGCCGAGGGATATACTACGGATCCTACACCTTCTTAGAAAC**

**ATGAAACATTGGAATTATCCTACTATTTGCAGTCATAGCAACCGCATTCATAGGTTATGT**

**ACTCCCA**

**>E2074.9f**

**TTTTGGCTCTCTTCTAGGAGTATGCCTTATAATTCAAATTATCACAGGCTTGTTTCTAGC**

**AATACACTACACATCCGACACTTTAACAGCATTCTCATCAGTTACTCACATCTGCCGAGA**

**CGTAAACTACGGCTGACTAATCCGATACTTACATGCCAACGGAGCCTCAATATTCTTTAT**

**CTGCTTATTCCTCCATGTAGGCCGAGGGATATACTACGGATCCTACACCTTCTTAGAAAC**

**ATGAAACATTGGAATTATCCTACTATTTGCAGTCATAGCAACCGCATTCATAGGTTATGT**

**ACTCCCA**

**>E2074.9r**

**TTTTGGCTCTCTTCTAGGAGTATGCCTTATAATTCAAATTATCACAGGCTTGTTTCTAGC**

**AATACACTACACATCCGACACTTTAACAGCATTCTCATCAGTTACTCACATCTGCCGAGA**

**CGTAAACTACGGCTGACTAATCCGATACTTACATGCCAACGGAGCCTCAATATTCTTTAT**

**CTGCTTATTCCTCCATGTAGGCCGAGGGATATACTACGGATCCTACACCTTCTTAGAAAC**

**ATGAAACATTGGAATTATCCTACTATTTGCAGTCATAGCAACCGCATTCATAGGTTATGT**

**ACTCCCA**

**>E2074.11f**

**TTTTGGCTCTCTTCTAGGAGTATGCCTTATAATTCAAATTATCACAGGCTTGTNTCTAGC**

**AATACACTACACATCCGACACTTTAACAGCATTCTCATNANTTACTCACATCTGCCGAGA**

**CGTAAACTACGGCTGACTAATCCGATACTTACATGCCAACGGAGCCTCAATATTCTTTAT**

**CTGCTTATTCCTCCATGTAGGCCGAGGGATATACTACGGATCCTACACCTTCTTAGAAAC**

**ATGAAACATTGGAATTATCCTACTATTTGCAGTCATAGCAACCGCATTCATAGGTTATGT**

**ACTCCCA**

**>E2074.11r**

**TTTTGGCTCTCTTCTAGGAGTATGCCTTATAATTCAAATTATCACAGGCTTGTTTCTAGC**

**AATACACTACACATCCGACACTTTAACAGCATTCTCATCAGTTACTCACATCTGCCGAGA**

**CGTAAACTACGGCTGACTAATCCGATACTTACATGCCAACGGAGCCTCAATATTCTTTAT**

**CTGCNTATTCCTCCATGTAGGCCGAGGGATATACTACGGATCCTACACCTTCTTAGAAAC**

**ATGAAACATTGGAATTATCCTACTATTTGCAGTCATAGCAACCGCATTCATAGGTTATGT**

**ACTCCCA**

**>E2074.12f**

**TTTTGGCTCTCTTCTAGGAGTATGCCTTATAATTCAAATTATCACAGGCTTGTNTCTAGC**

**AATACACTACACATCCGACACTTTAACAGCATTCTCATCAGTTACTCACATCTGCCGAGA**

**CGTAAACTACGGCTGACTAATCNTNTNCTTACATGCCAACGGAGCCTCAATATTCTTTAT**

**CTGCTTATTCCTCCATGTAGGCCGAGGGATATACTACGGATCCTACACCTTCTTAGAAAC**

**ATGAAACATTGGAATTATCCTACTATTTGCAGTCATAGCAACCGCATTCATAGGTTATGT**

**ACTCCCA**

**>E2074.12r**

**TTTTGGCTCTCTTCTAGGAGTATGCCTTATAATTCAAATTATCACAGGCTTGTTTCTAGC**

**AATACACTACACATCCGACACTTTAACAGCATTCTCATCAGTTACTCACATCTGCCGAGA**

**CGTAAACTACGGCTGACTAATCCGATACTTACATGCCAACGGAGCCTCAATATTCTTTAT**

**CTGCTTATTCCTCCATGTAGGCCGAGGGATATACTACGGATCCTACACCTTCTTAGAAAC**

**ATGAAACATTGGAATTATCCTACTATTTGCAGTCATAGCAACCGCATTCATAGGTTATGT**

**ACTCCCA**

**>**

**>E2075.1f**

**TTTTGGCTCTCTTCTAGGAGTATGCCTTATAATTCAAATTATCACAGGCTTGTTTCTAGC**

**AATACACTACACATCCGACACTTTAACAGCATTCTCATCAGTTACTCACATCTGCCGAGA**

**CGTAAACTACGGCTGACTAATCCGATACTTACATGCCAACGGAGCCTCAATATTCTTTAT**

**CTGCTTATTCCTCCATGTAGGCCGAGGGATATACTACGGATCCTACACCTTCTTAGAAAC**

**ATGAAACATTGGAATTATCCTACTATTTGCAGTCATAGCAACCGCATTCATAGGTTATGT**

**ACTCCCA**

**>E2075.1r**

**TTTTGGCTCTCTTCTAGGAGTATGCCTTATAATTCAAATTATCACAGGCTTGTNTCTAGC**

**AATACACTACACATCCGACACTTTAACAGCATTCTCATCAGTTACTCACATCTGCCGAGA**

**CGTAAACTACGGCTGACTAATCCGATACTTACATGCCAACGGAGCCTCAATATTCTTTAT**

**CTGCTTATTCCTCCATGTAGGCCGAGGGATATACTACGGATCCTACACCTTCTTAGAAAC**

**ATGAAACATTGGAATTATCCTACTATTTGCAGTCATAGCAACCGCATTCATAGGTTATGT**

**ACTCCCA**

**>E2075.2f**

**TTTTGGCTCTCTTCTAGGAGTATGCCTTATAATTCAAATTATCACAGGCTTGTTTCTAGC**

**AATACACTACACATCCGACACTTTAACAGCATTCTCATCAGTTACTNGCATCTGCCGAGA**

**CGTAAACTACGGCTGACTAATCCGATACTTACATGCCAACGNAANAAAAATATTCTTTAT**

**CTGCTTATTCCTCCATGTAGGCCGAGGGAAAAAATACGGATCCTACACCTTCTTAGAAAC**

**ATGAAACATTGGAATTATCCTACTATTTGCAGTCATAGCAACCGCATTCATAGGTTATGT**

**ACTCCCA**

**>E2075.2r**

**TTTTGGCTCTCTTCTAGGAGTATGCCTTATAATTCAAATTATCACAGGCTTGTNTCTAGC**

**AATACACTACACATCCGACACTTTAACAGCATTCTCATCAGTTACTCACATCTGCCGAGA**

**CGTAAACTACGGCTGACTAATCCGATACTTACATGCCAACGGAGCCTCAATATTCTTTAT**

**CTGCTTATTCCTCCATGTAGGCCGAGGGATATACTACGGATCCTACACCTTCTTAGAAAC**

**ATGAAACATTGGAATTATCCTACTATTTGCAGTCATAGCAACCGCATTCATAGGTTATGT**

**ACTCCCA**

**>E2075.3f**

**TTTTGGCTCTCTTCTAGGAGTATGCCTTATAATTCAAATTATCACAGGCTTGTTTCTAGC**

**AATACACTACACATCCGACACTTTAACAGCATTCTCATCAGTTACTCACATCTGCCGAGA**

**CGTAAACTACGGCTGACTAATCCGATACTTACATGCCAACGGAGCCTCAATATTCTTTAT**

**CTGCTTATTCCTCCATGTAGGCCGAGGGATANANTACGGATCCTACACCTTCTTAGAAAC**

**ATGAAACATTGGAATTATCCTACTATTTGCAGTCATAGCAACCGCATTCATAGGTTATGT**

**ACTCCCA**

**>E2075.3r**

**TTTTGGCTCTCTTCTAGGAGTATGCCTTATAATTCAAATTATCACAGGCTTGTNTCTAGC**

**AATACACTACACATCCGACACTTTAACAGCATTCTCATCAGTTACTCACATCTGCCGAGA**

**CNTAAACTACGGCTGACTAATCCGATACTTACATGCCAACGGAGCCTCAATATTCTTTAT**

**CTGCTTATTCCTCCATGTNGGCCGAGGGATATACTACGGATCCTACACCTTCTTAGAAAC**

**ATGAAACATTGGAATTATCCTACTATTTGCAGTCATAGCAACCGCATTCATAGGTTATGT**

**ACTCCCA**

**>E2075.4f**

**TTTTGGCTCTCTTCTAGGAGTATGCCTTATAATTCAAATTATCACAGGCTTGTTTCTAGC**

**AATACACTACACATCCGACACTTTAACAGCATTCTCATCAGTTACTCACATCTGCCGAGA**

**CGTAAACTACGGCTGACTAATCCGGTACTTACATGCCAACGGAGCCTCAATATTCTTTAT**

**CTGCTTATTCCTCCATGTAGGCCGAGGGATATACTACGGATCCTACACCTTCTTAGAAAC**

**ATGAAACATTGGAATTATCCTACTATTTGCAGTCATAGCAACCGCATTCATAGGTTATGT**

**ACTCCCA**

**>E2075.4r**

**TTTTGGCTCTCTTCTAGGAGTATGCCTTATAATTCAAATTATCACAGGCTTGTTTCTAGC**

**AATACACTACACATCCGACACTTTAACAGCATTCTCATCAGTTACTCACATCTGCCGAGA**

**CGTAAACTACGGCTGACTAATCCGGTACTTACATGCCAACGGAGCCTCAATATTCTTTAT**

**CTGCTTATTCCTCCATGTAGGCCGAGGGATATACTACGGATCCTACACCTTCTTAGAAAC**

**ATGAAACATTGGAATTATCCTACTATTTGCAGTCATAGCAACCGCATTCATAGGTTATGT**

**ACTCCCA**

**>E2075.5f**

**TTTTGGCTCTCTTCTAGGAGTATGCCTTATAATTCAAATTATCACAGGCTTGTTTCTAGC**

**AATACACTACACATCCGACACTTTAACAGCATTCTCATCAGTTACTCACATCTGCCGAGA**

**CGTAAACTACGGCTGACTAATCCGATACTTACATGCCAACGGAGCCTCAATATTCTTTAT**

**CTGCTTATTCCTCCATGTAGGCCGAGGGATATANTACGGATCCTACACCTTCTTAGAAAC**

**ATGAAACATTGGAATTATCCTACTATTTGCAGTCATAGCAACCGCATTCATAGGTTATGT**

**ACTCCCA**

**>E2075.5r**

**TTTTGGCTCTCTTCTAGGAGTATGCCTTATAATTCAAATTATCACAGGCTTGTNTCTAGC**

**AATACACTACACATCCGACACTTTAACAGCATTCTCATCAGTTACTCACATCTGCCGAGA**

**CNTAAACTACGGCTGACTAATCCGATACTTACATGCCAACGGAGCCTCAATATTCTTTAT**

**CTGCTTATTCCTCCATGTAGGCCGAGGGATATACTACGGATCCTACACCTTCTTAGAAAC**

**ATGAAACATTGGAATTATCCTACTATTTGCAGTCATAGCAACCGCATTCATAGGTTATGT**

**ACTCCCA**

**>**

**>18606.7f**

**TTTTGGCTCTCTTCTAGGAGTATGCCTTATAATTCAAATTATCACAGGCTTGTTTCTAGC**

**AATACACTACACATCCGACACTTTAACAGCATTCTCATCAGTTACTCACATCTGCCGAGA**

**CGTAAACTACGGCTGACTAATCCGATACTTACATGCCAACGNANNCTCAATATTCTTTAT**

**CTGCTTATTCCTCCATGTAGGCCGAGGGATATACTACGGATCCTACACCTTCTTAGAAAC**

**ATGAAACATTGGAATTATCCTACTATTTGCAGTCATAGCAACCGCATTCATAGGTTATGT**

**ACTCCCA**

**>18606.7r**

**TTTTGGCTCTCTTCTAGGAGTATGCCTTATAATTCAAATTATCACAGGCTTGTTTCTAGC**

**AATACACTACACATCCGACACTTTAACAGCATTCTCATCAGTTACTCACATCTGCCGAGA**

**NNTNAACTACGGCTGACTAATCCGATACTTACATGCCAACGGAGCCTCAATATTCTTTAT**

**CTGCTTATTCCTCCATGTAGGCCGAGGGATATACTACGGATCCTACACCTTCTTAGAAAC**

**ATGAAACATTGGAATTATCCTACTATTTGCAGTCATAGCAACCGCATTCATAGGTTATGT**

**ACTCCCA**

**>18606.8f**

**TTTTGGCTCTCTTCTAGGAGTATGCCTTATAATTCAAATTATCACAGGCTTGTNTCTAGC**

**AATACACTACACATCCGACACTTTAACAGCATTCTCATCAGTTACTCACATCTGCCGAGA**

**CGTAAACTACGGCTGACTAATCCGATACTTACATGCCAACGGAGCCTCAATATTCTTTAT**

**CTGCTTATTCCTCCATGTAGGCCGAGGGATATACTACGGATCCTACACCTTCTTAGAAAC**

**ATGAAACATTGGAATTATCCTACTATTTGCAGTCATAGCAACCGCATTCATAGGTTATGT**

**ACTCCCA**

**>18606.8r**

**TTTTGGCTCTCTTCTAGGAGTATGCCTTATAATTCAAATTATCACAGGCTTGTTTCTAGC**

**AATACACTACACATCCGACACTTTAACAGCATTCTCATCAGTTACTCACATCTGCCGAGA**

**CGTAAACTACGGCTGACTAATCCGATACTTACATGCCAACGGAGCCTCAATATTCTTTAT**

**CTGCTTATTCCTCCATGTAGGCCGAGGGATATACTACGGATCCTACACCTTCTTAGAAAC**

**ATGAAACATTGGAATTATCCTACTATTTGCAGTCATAGCAACCGCATTCATAGGTTATGT**

**ACTCCCA**

**>18606.9f**

**TTTTGGCTCTCTTCTAGGAGNATGCCTTATAATTCAAATTATCACAGGCTTGTNTCTAGC**

**AATACACTACACATCCGACACTTTAACAGCATTCTCATCAGTTACTCACATCTGCCGAGA**

**CGTAAACTACGGCTGACTAATCCGATACTTACATGCCAACGGAGCCTCAATATTCTTTAT**

**CTGCNNATTCCTCCATGTAGGCCGAGGGATATACTACGGATCCTACACCTTCTTAGAAAC**

**ATGAAACATTGGAATTATCCTACTATTTGCAGTCATAGCAACCGCATTCATAGGTTATGT**

**ACTCCCA**

**>18606.9r**

**TTTTGGCTCTCTTCTAGGAGTATGCCTTATAATTCAAATTATCACAGGCTTGTTTCTAGC**

**AATACACTACACATCCGACACTTTAACAGCATTCTCATCAGTTACTCACATCTGCCGAGA**

**CGTAAACTACGGCTGACTAATCCGATACTTACATGCCAACGGAGCCTCAATATTCTTTAT**

**CTGCNTANTCCTCCATGTAGGCCGAGGGATATACTACGGATCCTACACCTTCTTAGAAAC**

**ATGAAACATTGGAATTATCCTACTATTTGCAGTCATAGCAACCGCATTCATAGGTTATGT**

**ACTCCCA**

**>18606.10f**

**TTTTGGCTCTCTTCTAGGAGTATGCCTTATAATTCAAATTATCACAGGCTTGTTTCTAGC**

**AATACACTACACATCCGACACTTTAACAGCATTCTCATCAGTTACTCACATCTGCCGAGA**

**CGTAAACTACGGCTGACTAATCCGATACTTACATGCCAACGGAGCCTCAATATTCTTTAT**

**CTGCTTATTCCTCCATGTAGGCCGAGGGATATACTACGGATCCTACACCTTCTTAGAAAC**

**ATGAAACATTGGAATTATCCTACTATTTGCAGTCATAGCGACCGCATTCATAGGTTATGT**

**ACTCCCA**

**>18606.10r**

**TTTTGGCTCTCTTCTAGGAGTATGCCTTATAATTCAAATTATCACAGGCTTGTTTCTAGC**

**AATACACTACACATCCGACACTTTAACAGCATTCTCATCAGTTACTCACATCTGCCGAGA**

**CGTAAACTACGGCTGACTAATCCGATACTTACATGCCAACGGAGCCTCAATATTCTTTAT**

**CTGCTTATTCCTCCATGTAGGCCGAGGGATATACTACGGATCCTACACCTTCTTAGAAAC**

**ATGAAACATTGGAATTATCCTACTATTTGCAGTCATAGCGACCGCATTCATAGGTTATGT**

**ACTCCCA**

**>18606.12f**

**TTTTGGCTCTCTTCTAGGAGTATGCCTTATAATTCAAATTATCACAGGCTTGTNTCTAGC**

**AATACACTACACATCCGACACTTTAACAGCATTCTCATCAGTTACTCACATCTGCCGAGA**

**CGTAAACTACGGCTGACTAATCCGATACTTACATGCCAACGGAGCCTCAATATTCTTTAT**

**CTGCTTATTCCTCCATGTAGGCCGAGGGATATACTACGGATCCTACACCTTCTTAGAAAC**

**ATGAAACATTGGAATTATCCTACTATTTGCAGTCATAGCAACCGCATTCATAGGTTATGT**

**ACTCCCA**

**>18606.12r**

**TTTTGGCTCTCTTCTAGGAGTATGCCTTATAATTCAAATTATCACAGGCTTGTTTCTAGC**

**AATACACTACACATCCGACACTTTAACAGCATTCTCATCAGTTACTCACATCTGCCGAGA**

**CGTAAACTACGGCTGACTAATCCGATACTTACATGCCAACGGAGCCTCAATATTCTTTAT**

**CNGAANANTCCTCCATGTAGGCCGAGGGATATACTACGGATCCTACACCTTCTTAGAAAC**

**ATGAAACATTGGAATTATCCTACTATTTGCAGTCATAGCAACCGCATTCATAGGTTATGT**

**ACTCCCA**

**>**

**>18607.5f**

**TTTTGGCTCTCTTCTAGGAGTATGCCTTATAGTTCAAATTATCACAGGATTATTTCTAGC**

**AATACACTACACATCCGACACTTTAACAGCATTCTCATCAGTTACTCACATCTGCCGAGA**

**CGTAAACTACGGCTGACTAATCCGATACTTACATGCCAACGGAGCCTCAATATTCTTTAT**

**CTGCTTATTCCTCCATGTAGGCCGAGGGATATACTACGGATCCTACACCTTCTTAGAAAC**

**ATGAAACATTGGAATTATCCTACTATTTGCAGTCATAGCAACCGCATTCATAGGTTATGT**

**ACTCCCA**

**>18607.5r**

**TTTTGGCTCTCTTCTAGGAGTATGCCTTATAGTTCAAATTATCACAGGATTATTTCTAGC**

**AATACACTACACATCCGACACTTTAACAGCATTCTCATCAGTTACTCACATCTGCCGAGA**

**CGTAAACTACGGCTGACTAATCCGATACTTACATGCCAACGGAGCCTCAATATTCTTTAT**

**CTGCTTATTCCTCCATGTAGGCCGAGGGATATACTACGGATCCTACACCTTCTTAGAAAC**

**ATGAAACATTGGAATTATCCTACTATTTGCAGTCATAGCAACCGCATTCATAGGTTATGT**

**ACTCCCA**

**>18607.6f**

**TTTTGGCTCTCTTCTAGGAGTATGCCTTATAGTTCAAATTATCACAGGATTATTTCTAGC**

**AATACACTACACATCCGACACTTTAACAGCATTCTCATCAGTTACTCACATCTGCCGAGA**

**CGTAAACTACGGCTGACTAATCCGATACTTACATGCCAACGGAGCCTCAATATTCTTTAT**

**CTGCTTATTCCTCCATGTAGGCCGAGGGATATACTACGGATCCTACACCTTCTTAGAAAC**

**ATGAAACATTGGAATTATCCTACTATTTGCAGTCATAGCAACCGCATTCATAGGTTATGT**

**ACTCCCA**

**>18607.7f**

**TTTTGGCTCTCTTCTAGGAGTATGCCTTATAGTTCAAATTATCACAGGATTATTTCTAGC**

**AATACACTACACATCCGACACTTTAACAGCATTCTCATCAGTTACTCACATCTGCCGAGA**

**CGTAAACTACGGCTGACTAATCCGATACTTACATGCCAACGGAGCCTCAATATTCTTTAT**

**CTGCTTATTCCTCCATGTAGGCCGAGGGATATACTACGGATCCTACACCTTCTTAGAAAC**

**ATGAAACATTGGAATTATCCTACTATTTGCAGTCATAGCAACCGCATTCATAGGTTATGT**

**ACTCCCA**

**>18607.7r**

**TTTTGGCTCTCTTCTAGGAGTATGCCTTATAGTTCAAATTATCACAGGATTATTTCTAGC**

**AATACACTACACATCCGACACTTTAACAGCATTCTCATCAGTTACTCACATCTGCCGAGA**

**CGTAAACTACGGCTGACTAATCCGATACTTACATGCCAACGGAGCCTCAATATTCTTTAT**

**CTGCTTATTCCTCCATGTAGGCCGAGGGATATACTACGGATCCTACACCTTCTTAGAAAC**

**ATGAAACATTGGAATTATCCTACTATTTGCAGTCATAGCAACCGCATTCATAGGTTATGT**

**ACTCCCA**

**>18607.8f**

**TTTTGGCTCTCTTCTAGGAGTATGCCTTATAGTTCAAATTATCACAGGATTATTTCTAGC**

**AATACACTACACATCCGACACTTTAACAGCATTCTCATCAGTTACTCACATCTGCCGAGA**

**CGTAAACTACGGCTGACTAATCCGATACTTACATGCCAACGGAGCCTCAATATTCTTTAT**

**CTGCTTATTCCTCCATGTAGGCCGAGGGATATACTACGGATCCTACACCTTCTTAGAAAC**

**ATGAAACATTGGAATTATCCTACTATTTGCAGTCATAGCAACCGCATTCATAGGTTATGT**

**ACTCCCA**

**>18607.8r**

**TTTTGGCTCTCTTCTAGGAGTATGCCTTATAGTTCAAATTATCACAGGATTATTTCTAGC**

**AATACACTACACATCCGACACTTTAACAGCATTCTCATCAGTTACTCACATCTGCCGAGA**

**CGTAAACTACGGCTGACTAATCCGATACTTACATGCCAACGGAGCCTCAATATTCTTTAT**

**CTGCTTATTCCTCCATGTAGGCCGAGGGATATACTACGGATCCTACACCTTCTTAGAAAC**

**ATGAAACATTGGAATTATCCTACTATTTGCAGTCATAGCAACCGCATTCATAGGTTATGT**

**ACTCCCA**

**>18607.9f**

**TTTTGGCTCTCTTCTAGGAGTATGCCTTATAGTTCAAATTATCACAGGATTATTTCTAGC**

**AATACACTACACATCCGACACTTTAACAGCATTCTCATCAGTTACTCACATCTGCCGAGA**

**CGTAAACTACGGCTGACTAATCCGATACTTACATGCCAACGGAGCCTCAATATTCTTTAT**

**CTGCTTATTCCTCCATGTAGGCCGAGGGATATACTACGGATCCTACACCTTCTTAGAAAC**

**ATGAAACATTGGAATTATCCTACTATTTGCAGTCATAGCAACCGCATTCATAGGTTATGT**

**ACTCCCA**

**>18607.9r**

**TTTTGGCTCTCTTCTAGGAGTATGCCTTATAGTTCAAATTATCACAGGATTATTTCTAGC**

**AATACACTACACATCCGACACTTTAACAGCATTCTCATCAGTTACTCACATCTGCCGAGA**

**CGTAAACTACGGCTGACTAATCCGATACTTACATGCCAACGGAGCCTCAATATTCTTTAT**

**CTGCTTATTCCTCCATGTAGGCCGAGGGATATACTACGGATCCTACACCTTCTTAGAAAC**

**ATGAAACATTGGAATTATCCTACTATTTGCAGTCATAGCAACCGCATTCATAGGTTATGT**

**ACTCCCA**

**>**

**>18608.2f**

**TTTTGGCTCTCTTCTAGGAGTATGCCTTATAATTCAAATTATCACAGGCTTGTNTCTAGC**

**AATACACTACACATCCGACACTTTAACAGCATTCTCATCAGTTACTCACATCTGCCGAGA**

**CGTAAACTACGGCTGACTAATCCGATACTTACATGCCAGCGGAGCCTCAATATTCTTTAT**

**CTGCTTATTCCTCCATGTAGGCCGAGGGATATACTACGGATCCTACACCTTCTTAGAAAC**

**ATGAAACATTGGAATTATCCTACTATTTGCAGTCATAGCAACCGCATTCATAGGTTATGT**

**ACTCCCA**

**>18608.2r**

**TTTTGGCTCTCTTCTAGGAGTATGCCTTATAATTCAAATTATCACAGGCTTGTTTCTAGC**

**AATACACTACACATCCGACACTTTAACAGCATTCTCATCAGTTACTCACATCTGCCGAGA**

**CGTAAACTACGGCTGACTAATCCGATACTTACATGCCAGCGGAGCCTCAATATTCTTTAT**

**CTGCTTATTCCTCCATGTAGGCCGAGGGATATACTACGGATCCTACACCTTCTTAGAAAC**

**ATGAAACATTGGAATTATCCTACTATTTGCAGTCATAGCAACCGCATTCATAGGTTATGT**

**ACTCCCA**

**>18608.3f**

**TTTTGGCTCTCTTCTAGGAGTATGCCTTATAATTCAAATTATCACAGGCTTGTTTCTAGC**

**AATACACTACACATCCGACACTTTAACAGCATTCTCATCAGTTACTCACATCTGCCGAGA**

**CGTAAACTACGGCTGACTAATCCGATACTTACATGCCAACGGAGCCTCAATATTCTTTAT**

**CTGCTTATTCCTCCATGTAGGCCGAGGGATATACTACGGATCCTACACCTTCTTAGAAAC**

**ATGAAACATTGGAATTATCCTACTATTTGCAGTCATAGCAACCGCATTCATAGGTTATGT**

**ACTCCCA**

**>18608.3r**

**TTTTGGCTCTCTTCTAGGAGTATGCCTTATAATTCAAATTATCACAGGCTTGTTTCTAGC**

**AATACACTACACATCCGACACTTTAACAGCATTCTCATCAGTTACTCACATCTGCCGAGA**

**CGTAAACTACGGCTGACTAATCCGATACTTACATGCCAACGGAGCCTCAATATTCTTTAT**

**CTGCTTATTCCTCCATGTAGGCCGAGGGATATACTACGGATCCTACACCTTCTTAGAAAC**

**ATGAAACATTGGAATTATCCTACTATTTGCAGTCATAGCAACCGCATTCATAGGTTATGT**

**ACTCCCA**

**>18608.4f**

**TTTTGGCTCTCTTCTAGGAGTATGCCTTATAATTCAAATTATCACAGGCTTGTTTCTAGC**

**AATACACTACACATCCGACACTTCAACAGCATTCTCATCAGTTACTCACATCTGCCGAGA**

**CGTAAACTACGGCTGACTAATCCGATACTTACATGCCAACGGAGCCTCAATATTCTTTAT**

**CTGCTTATTCCTCCATGTAGGCCGAGGGATATACTACGGATCCTACACCTTCTTAGAAAC**

**ATGAAACATTGGAATTATCCTACTATTTGCAGTCATAGCAACCGCATTCATAGGTTATGT**

**ACTCCCA**

**>18608.4r**

**TTTTGGCTCTCTTCTAGGAGTATGCCTTATAATTCAAATTATCACAGGCTTGTTTCTAGC**

**AATACACTACACATCCGACACTTCAACAGCATTCTCATCAGTTACTCACATCTGCCGAGA**

**CGTAAACTACGGCTGACTAATCCGATACTTACATGCCAACGGAGCCTCAATATTCTTTAT**

**CTGCTTATTCCTCCATGTAGGCCGAGGGATATACTACGGATCCTACACCTTCTTAGAAAC**

**ATGAAACATTGGAATTATCCTACTATTTGCAGTCATAGCAACCGCATTCATAGGTTATGT**

**ACTCCCA**

**>18608.5f**

**TTTTGGCTCTCTTCTAGGAGTATGCCTTATAATTCAAATTATCACAGGCTTGTNTCTAGC**

**AATACACTACACATCCGACACTTTAACAGCATTCTCATCAGTTACTCACATCTGCCGAGG**

**CGTAAACTACGGCTGACTAATCCGATACTTACATGCCAACGGAGCCTCAATATTCTTTAT**

**CTGCTTATTCCTCCATGTAGGCCGAGGGATATACTACGGATCCTACACCTTCTTAGAAAC**

**ATGAAACATTGGAATTATCCTACTATTTGCAGTCATAGCAACTGCATTCATAGGTTATGT**

**ACTCCCA**

**>18608.5r**

**TTTTGGCTCTCTTCTAGGAGTATGCCTTATAATTCAAATTATCACAGGCTTGTTTCTAGC**

**AATACACTACACATCCGACACTTTAACAGCATTCTCATCAGTTACTCACATCTGCCGAGG**

**CGTAAACTACGGCTGACTAATCCGATACTTACATGCCAACGGAGCCTCAATATTCTTTAT**

**CTGCTTATTCCTCCATGTAGGCCGAGGGATATACTACGGATCCTACACCTTCTTAGAAAC**

**ATGAAACATTGGAATTATCCTACTATTTGCAGTCATAGCAACTGCATTCATAGGTTATGT**

**ACTCCCA**

**>18608.8f**

**TTTTGGCTCTCTTCTAGGAGTATGCCTTATAATTCAAATTATCACAGGCTTGTTTCTAGC**

**AATACACTACACATCCGACACTTTAACAGCATTCTCATCAGTTACTCACATCTGCCGAGA**

**CGTAAACTACGGCTGACTAATCCGATACTTACATGCCAACGGAGCCTCAATATTCTTTAT**

**CTGCTTATTCCTCCATGTAGGCCGAGAGATATACTACGGATCCTACACCTTCTTAGAAAC**

**ATGAAACATTGGAATTATCCTACTATTTGCAGTCATAGCAACCGCATTCATAGGTTATGT**

**ACTCCCA**

**>18608.8r**

**TTTTGGCTCTCTTCTAGGAGTATGCCTTATAATTCAAATTATCACAGGCTTGTTTCTAGC**

**AATACACTACACATCCGACACTTTAACAGCATTCTCATCAGTTACTCACATCTGCCGAGA**

**CGTAAACTACGGCTGACTAATCCGATACTTACATGCCAACGGAGCCTCAATATTCTTTAT**

**CTGCTTATTCCTCCATGTAGGCCGAGAGATATACTACGGATCCTACACCTTCTTAGAAAC**

**ATGAAACATTGGAATTATCCTACTATTTGCAGTCATAGCAACCGCATTCATAGGTTATGT**

**ACTCCCA**

**>**

**>18842.1f**

**TTTTGGCTCTCTTCTAGGAGTATGCCTTATAGTTCAAATTATCACAGGATTATTTCTAGC**

**AATACACTACACATCCGACACTTTAACAGCATTCTCATCAGTTACTCACATCTGCCGAGA**

**CGTAAACTACGGCTGACTAATCCGATACTTACATGCCAACGGAGCCTCAATATTCTTTAT**

**CTGCTTATTCCTCCATGTAGGCCGAGGGATATACTACGGATCCTACACCTTCTTAGAAAC**

**ATGAAACATTGGAATTATCCTACTATTTGCAGTCATAGCAACCGCATTCATAGGTTATGT**

**ACTCCCA**

**>18842.1r**

**TTTTGGCTCTCTTCTAGGAGTATGCCTTATAGTTCAAATTATCACAGGATTATTTCTAGC**

**AATACACTACACATCCGACACTTTAACAGCATTCTCATCAGTTACTCACATCTGCCGAGA**

**CGTAAACTACGGCTGACTAATCCGATACTTACATGCCAACGGAGCCTCAATATTCTTTAT**

**CTGCTTATTCCTCCATGTAGGCCGAGGGATATACTACGGATCCTACACCTTCTTAGAAAC**

**ATGAAACATTGGAATTATCCTACTATTTGCAGTCATAGCAACCGCATTCATAGGTTATGT**

**ACTCCCA**

**>18842.4f**

**TTTTGGCTCTCTTCTAGGAGTATGCCTTATAGTTCAAATTATCACAGGATTATTTCTAGC**

**AATACACTACACATCCGACACTTTAACAGCATTCTCATCAGTTACTCACATCTGCCGAGA**

**CGTAAACTACGGCTGACTAATCCNATNCTTACATGCCAACGGAGCCTCAATATTCTTTAT**

**CTGCTTATTCCTCCATGTAGGCCGAGGGATATACTACGGATCCTACACCTTCTTAGAAAC**

**ATGAAACATTGGAATTATCCTACTATTTGCAGTCATAGCAACCGCATTCATAGGTTATGT**

**ACTCCCA**

**>18842.4r**

**TTTTGGCTCTCTTCTAGGAGTATGCCTTATAGTTCAAATTATCACAGGATTATTTCTAGC**

**AATACACTACACATCCGACACTTTAACAGCATTCTCATCAGTTACTCACATCTGCCGAGA**

**CGTAAACTACGGCTGACTAATCCGATACTTACATGCCAACGGAGCCTCAATATTCTTTAT**

**CTGCTTATTCCTCCATGTAGGCCGAGGGATATACTACGGATCCTACACCTTCTTAGAAAC**

**ATGAAACATTGGAATTATCCTACTATTTGCAGTCATAGCAACCGCATTCATAGGTTATGT**

**ACTCCCA**

**>18842.5f**

**TTTTGGCTCTCTTCTAGGAGTATGCCTTATAGTTCAAATTATCACAGGATTATTTCTAGC**

**AATACACTACACATCCGACACTTTAACAGCATTCTCATCAGTTACTCACATCTGCCGAGA**

**CGTAAACTACGGCTGACTAATNNNATACTTACATGCCAACGGAGCCTCAATATTCTTTAT**

**CTGCTTATTCCTCCATGTAGGCCGAGGGATATACTACGGATCCTACACCTTCTTAGAAAC**

**ATGAAACATTGGAATTATCCTACTATTTGCAGTCATAGCAACCGCATTCATAGGTTATGT**

**ACTCCCA**

**>18842.5r**

**TTTTGGCTCTCTTCTAGGAGTATGCCTTATAGTTCAAATTATCACAGGATTATTTCTAGC**

**AATACACTACACATCCGACACTTTAACAGCATTCTCATCAGTTACTCACATCTGCCGAGA**

**CGTAAACTACGGCTGACTAATCCGATACTTACATGCCAACGGAGCCTCAATATTCTTTAT**

**CTGCTTATTCCTCCATGTAGGCCGAGGGATATACTACGGATCCTACACCTTCTTAGAAAC**

**ATGAAACATTGGAATTATCCTACTATTTGCAGTCATAGCAACCGCATTCATAGGTTATGT**

**ACTCCCA**

**>18842.6f**

**TTTTGGCTCTCTTCTAGGAGTATGCCTTATAGTTCAAATTATCACAGGATTATTTCTAGC**

**AATACACTACACATCCGACACTTTAACAGCATTCTCATCAGTTACTCACATCTGCCGAGA**

**CGTAAACTACGGCTGACTAATCCNATNCTTACATGCCAACGGAGCCTCAATATTCTTTAT**

**CTGCTTATTCCTCCATGTAGGCCGAGGGATATACTACGGATCCTACACCTTCTTAGAAAC**

**ATGAAACATTGGAATTATCCTACTATTTGCAGTCATAGCAACCGCATTCATAGGTTATGT**

**ACTCCCA**

**>18842.6r**

**TTTTGGCTCTCTTCTAGGAGTATGCCTTATAGTTCAAATTATCACAGGATTATTTCTAGC**

**AATACACTACACATCCGACACTTTAACAGCATTCTCATCAGTTACTCACATCTGCCGAGA**

**CGTAAACTACGGCTGACTAATCCGATACTTACATGCCAACGGAGCCTCAATATTCTTTAT**

**CTGCTTATTCCTCCATGTAGGCCGAGGGATATACTACGGATCCTACACCTTCTTAGAAAC**

**ATGAAACATTGGAATTATCCTACTATTTGCAGTCATAGCAACCGCATTCATAGGTTATGT**

**ACTCCCA**

**>18842.9f**

**TTTTGGCTCTCTTCTAGGAGTATGCCTTATAGTTCAAATTATCACAGGATTATTTCTAGC**

**AATACACTACACATCCGACACTTTAACAGCATTCTCATCAGTTACTCACATCTGCCGAGA**

**CGTAAACTACGGCTGACTAATCCGATACTTACATGCCAACGGAGCCTCAATATTCTTTAT**

**CTGCTTATTCCTCCATGTAGGCCGAGGGATATACTACGGATCCTACACCTTCTTAGAAAC**

**ATGAAACATTGGAATTATCCTACTATTTGCAGTCATAGCAACCGCATTCATAGGTTATGT**

**ACTCCCA**

**>18842.9r**

**TTTTGGCTCTCTTCTAGGAGTATGCCTTATAGTTCAAATTATCACAGGATTATTTCTAGC**

**AATACACTACACATCCGACACTTTAACAGCATTCTCATCAGTTACTCACATCTGCCGAGA**

**CGTAAACTACGGCTGACTAATCCGATACTTACATGCCAACGGAGCCTCAATATTCTTTAT**

**CTGCTTATTCCTCCATGTAGGCCGAGGGATATACTACGGATCCTACACCTTCTTAGAAAC**

**ATGAAACATTGGAATTATCCTACTATTTGCAGTCATAGCAACCGCATTCATAGGTTATGT**

**ACTCCCA**

**>**

**>RAG A**

**>2074A.5**

**CTTCAAGAGTGACAGGCACAACCGGAGATACCCAGTCCACGGGCCCGTGGACGCTAAAAC**

**>2074A.6**

**CTTCAAGAGTGACAGGCACAACCGGAGATACCCAGTCCACGGGCCCGTGGACGCTAAAAC**

**>2074B.1.1**

**CTTCAAGAGTGACAGGCACAACCGGAGATACCCAGTCCACGGGCCCGTGGACGCTAAAAC**

**>2074B.1.2**

**CTTCAAGAGTGACAGGCACAACCGGAGATACCCAGTCCACGGGCCCGTGGACGCTAAAAC**

**>2074B.1.3**

**CTTCAAGAGTGACGGGCACAACCGGAGATACCCAGTCCACGGGCCCGTGGACGCTAAAAC**

**>**

**>2075A.4.6**

**CTTCAAGAGTGACAGGCACAACCGGAGATACCCAGTCCACGGGCCCGTGGACGCTAAAAC**

**>2075A.4.13**

**CTTCAAGAGTGACAGGCACAACCGGAGATACCCAGTCCACGGGCCCGTGGACGCTAAAAC**

**>2075B.5.11**

**CTTCAAGAGTGACGGGCACAACCGGAGATACCCAGTCCACGGGCCCGTGGACGCTAAAAC**

**>2075B.5.18**

**CTTCAAGAGTGACGGGCACAACCGGAGATACCCAGTCCACGGGCCCGTGGACGCTAAAAC**

**>2075C.33G.Denmark**

**CTTCAAGAGTGACAGGCACAACCGGAGATACCCAGTCCACGGGCCCGTGGACGCTAAAAC**

**>2075C.34G.Denmark**

**CTTCAAGAGTGACAGGCACAACCGGAGATACCCAGTCCACGGGCCCGTGGACGCTAAAAC**

**>2075C.35G.Denmark**

**CTTCAAGAGTGACAGGCACAACCGGAGATACCCAGTCCACGGGCCCGTGGACGCTAAAAC**

**>2075.D.Denmark_1-RAG**

**CTTCAAGAGTGACGGGCACAACCGGAGATACCCAGTCCACGGGCCCGTGGACGCTAAAAC**

**>2075.D.Denmark_1-RAG**

**CTTCAAGAGTGACGGGCACAACCGGAGATACCCAGTCCACGGGCCCGTGGACGCTAAAAC**

**>2075.D.Denmark_1-RAG**

**CTTCAAGAGTGACGGGCACAACCGGAGATACCCAGTCCACGGGCCCGTGGACGCTAAAAC**

**>2075.D.Denmark_1-RAG**

**CTTCAAGAGTGACAGGCACAACCGGAGATACCCAGTCCACGGGCCCGTGGACGCTAAAAC**

**>2075.D..Denmark_1-RAG**

**CTTCAAGAGTGACAGGCACAACCGGAGATACCCAGTCCACGGGCCCGTGGACGCTAAAAC**

**>**

**>18606A.1.1**

**CTTCAAGAGTGACAGGCACAACCGGAGATACCCAGTCCACGGGCCCGTGGACGCTAAAAC**

**>18606A.1.3**

**CTTCAAGAGTGACAGGCACAACCGGAGATACCCAGTCCACGGGCCCGTGGACGCTAAAAC**

**>18606B.2.4**

**CTTCAAGAGTGACAGGCACAACCGGAAATACCCAGTCCACGGGCCCGTGGACGCTAAAAC**

**>18606B.2.5**

**CTTCAAGAGTGACAGGCACAACCGGAGATACCCAGTCCACGGGCCCGTGGACGCTAAAAC**

**>18606B.2.6**

**CTTCAAGAGTGACGGGCACAACCGGAGATACCCAGTCCACGGGCCCGTGGACGCTAAAAC**

**>**

**>18607A.1**

**CTTCAAGAGTGACGGGCACAACCGGAGATACCCAGTCCACGGACCCGTGGACGCTAAAAC**

**>18607A.2**

**CTTCAAGAGTGACGGGCACAACCGGAGATACCCAGTCCACGGGCCCGTGGACGCTAAAAC**

**>18607A.3**

**CTTCAAGAGTGACGTGCACAACCGGAGATACCCAGTCCACGGGCCTGTGGACGCTAAAAC**

**>18607A.4**

**CTTCAAGAGTGACGGGCACAACCGGAGATACCCAGTCCACGGGCCCGTGGACGCTAAAAC**

**>18607A.5**

**CTTCAAGAGTGACGGGCACAACCGGAGATACCCAGTCCACGGGCCCGTGGACGCTAAAAC**

**>18607A.7**

**CTTCAAGAGTGACGGGCACAACCGGAGATACCCAGTCCACGGGCCCGTGGACGCTAAAAC**

**>18607A.8**

**CTTCAAGAGTGACGGGCACAACCGGAGATACCCAGTCCACGGGCCCGTGGACGCTAAAAC**

**>18607A.9**

**CTTCAAGAGTGACGGGCACAACCGGAGATACCCAGTCCACGGACCCGTGGACGCTAAAAC**

**>18607B.57G.Denmark**

**CTTCAAGAGTGATGGGCACAACCGGAGATACCCAGTCCACGGGCCCGTGGATGCTAAAAC**

**>18607B.61G.Denmark**

**CTTCAAGAGTGACAGGCACAACCGGAGATACCCAGTCCACGGGCCCGTGGACGCTAAAAC**

**>18607B.62G.Denmark**

**CTTCAAGAGTGACAGGCACAACCGGAGATACCCAGTCCACGGTCCCGTGGACGCTAAAAC**

**>**

**>18608A.1.1**

**CTTCAAGAGTGACAGGCACAACCGGAGATACCCAGTCCACGGGCCCGTGGACGCTAAAAC**

**>18608A.1.2**

**CTTCAAGAGTGACGGGCACAACCGGAGATACCCAGTCCACGGGCCCGTGGACGCTAAAAC**

**>18608A.1.3**

**CTTCAAGAGTGACAGGCACAACCGGAGATACCCAGTCCACGGGCCCGTGGACGCTAAAAC**

**>18608B.2.4**

**CTTCAAGAGTGACAGGCACAACCGGAGATACCCAGTCCACGGGCCCGTGGACGCTAAAAC**

**>18608B.2.5**

**CTTCAAGAGTGACAGGCACAACCGGAGATACCCAGTCCACGGGCCCGTGGACGCTAAAAC**

**>18608B.2.6**

**CTTCAAGAGTGACGGGCACAACCGGAGATACCCAGTCCACGGGCCCGTGGACGCTAAAAC**

**>**

**>18842A.3.7**

**CTTCAAGAGTGACAGGCACAACCGGAGATACCCAGTCCACGGGCCCGTGGACGCTAAAAC**

**>18842A.3.8**

**CTTCAAGAGTGACAGGCACAACCGGAGATACCCAGTCCACGGGCCCGTGGACGCTAAAAC**

**>18842A.3.9**

**CTTCAAGAGTGACAGGCACAACCGGAGATACCCAGTCCACGGGCCCGTGGACGCTAAAAC**

**>18842B.1.2**

**CTTCAAGAGTGACAGGCACAACCGGAGATACCCAGTCCACGGGCCCGTGGACGCTAAAAC**

**>18842B.1.3**

**CTTCAAGAGTGACAGGCACAACCGGAGATACCCAGTCCACGGGCCCGTGAACGCTAAAAC**

**>**

**>2079A.D1**

**CTTCAAGAGTGACAGGCACAACCGGAGATACCCAGTCCACGGGCCCGTGGACGCTAAAAC**

**>2079A.D2**

**CTTCAAGAGTGACAGGCACAACCGGAGATACCCAGTCCACGGGCCCGTGGACGCTAAAAC**

**>2079A.D3**

**CTTCAAGAGTGACAGGCACAACCGGAGATACCCAGTCCACGGGCCCGTGGACGCTAAAAC**

**>2079B.U2**

**CTTCAAGAGTGACAGGCACAACCGGAGATACCCAGTCCACGGGCCCGTGGACGCTAAAAC**

**>2079B.U4**

**CTTCAAGAGTGACAGGCACAACCGGAGATACCCAGTCCACGGGCCCGTGGACGCTAAAAC**

**>2079B.U5**

**CTTCAAGAGTGACAGGCACAACCGGAGATACCCAGTCCACGGGCCCGTGGGCGCTAAAAC**

**>2079C.G50.Denmark**

**CTTCAAGAGTGACAGGCACAACCGGAGATACCCAGTCCACGGGCCCGTGGACGCTAAAAC**

**>2079C.G51.Denmark**

**CTTCAAGAGTGACAGGCACAACCGGAGATACCCAGTCCACGGTCCCGTGGACGCTAAAAC**

**>2079C.G52.Denmark**

**CTTCAAGAGTGACAGGCACAACCGGAGATACCCAGTCCACGGGCCCGTGGACGCTAAAAC**

**>2079C.G53.Denmark**

**CTTCAAGAGTGACAGGCACAACCGGAGATACCCAGTCCACGGGCCCGTGGACGCTAAAAC**

**>2079C.G54.Denmark**

**CTTCAAGAGTGACAGGCACAACCGGAGATACCCAGTCCACGGGCCCGTGGACGCTAAAAC**

**>**

**>2072A.3.4**

**CTTCAAGAGTGACAGGCACAACCGGAGATACCCAGTCCACGGGCCCGTGGACGCTAAAAC**

**>2072A.3.5**

**CTTCAAGAGTGACAGGCACAACCGGAGATACCCAGTCCACGGGCCCGTGGACGCTAAAAC**

**>2072A.3.6**

**CTTCAAGAGTGACGGGCACAACCAGAAATACCCAGTCCACGGGCCCGTGGACGCTAAAAC**

**>2072B.1.1**

**CTTCAAGAGTGACAGGCACAACCGGAGATACCCAGTCCACGGGCCCGTGGACGCTAAAAC**

**>2072B.1.4**

**CTTCAAGAGTGACAGGCACAACCGGAGATACCCAGTCCACGGGCCCGTGGACGCTAAAAC**

**>2072B.1.5**

**CTTCAAGAGTGACAGGCACAACCGGAGATACCCAGTCCACGGGCCCGTGGACGCTAAAAC**

**>**

**>2073A.7.10**

**CTTCAAGAGTGACAGGCACAACCGGAGATACCCAGTCCACGGGCCCGTGGACGCTAAAAC**

**>2073A.7.11**

**CTTCAAGAGTGACAGGCACAACCGGAGATACCCAGTCCACGGGCCCGTGGACGCTAAAAC**

**>2073A.7.12**

**CTTCAAGAGTGACAGGCACAACCGGAGATACCCAGTCCATGGGCCCGTGGACGCTAAAAC**

**>2073B.1.1**

**CTTCAAGAGTGACAGGCACAACCGGAGATACCCAGTCCACGGGCCCGTGGATGCTAAAAC**

**>2073B.1.2**

**CTTCAAGAATGACAGGCACAACCGGAGATACCCAGTCCACGGGCCCGTGGACGCTAAAAC**

**>**

**>2076A.11.4**

**CTTCAAGAGTGACGGGCACAACCGGAGATACCCAGTCCACGGGCCCGTGGACGCTAAAAC**

**>2076A.11.5**

**CTTCAAGAGTGACGGGCACAACCGGAGATACCCAGTCCACGGGCCCGTGGACGCTAAAAC**

**>2076A.11.6**

**CTTCAAGAGTGACGGGCACAACCGGAGATACCCAGTCCACGGGCCCGTGGACGCTAAAAC**

**>2076B.12.7**

**CTTCAAGAGTGACGGGCACAACCGGAGATACCTAGTCCACGGGCCCGTGGACGCTAAAAC**

**>2076B.12.8**

**CTTCAAGAGTGACGGGCACAACCGGAGATACCTAGTCCACGGGCCCGTGGACGCTAAAAC**

**>2076B.12.9**

**CTTCAAGAGTGACGGGCACAACCGGAGATACCCAGTCCACGGGCCCGTGGACGCTAAAAC**

**>**

**>2078A.7**

**CTTCAAGAGTGACGGGCACAACCGGAGATACCCAGTCCACGGGCCCGTGGACGCTAAAAC**

**>2078A.8**

**CTTCAAGAGTGACGGGCACAACCGGAGATACCCAGTCCACAGGCCCGTGGACGCTAAAAC**

**>2078B.9.13**

**CTTCAAGAGTGACAGGCACAACCGGAGATACCCAGTCCACGGGCCCGTGGACGCTAAAAC**

**>2078B.9.14**

**CTTCAAGAGTGACAGGCACAACCGGAGATACCCAGTCCACGGGCCCGTGGACGCTAAAAC**

**>2078B.9.15**

**CTTCAAGAGTGACGGGCACAACCGGAGATACCCAGTCCACGGGCCCGTGGACGCTAAAAC**

**>**

**>2080A.11.12**

**CTTCAAGAGTGACAGGCACAACCGGAGATACCCAGTCCACGGGCCCGTGGACGCTAAAAC**

**>2080A.11.16**

**CTTCAAGAGTGACAGGCACAACCGGAGATACCCAGTCCACGGGCCCGTGGACGCTAAAAC**

**>2080A.11.17**

**CTTCAAGAGTGACAGGCACAACCGGAGATACCCAGTCCACGGGCCCGTGGACGCTAAAAC**

**>**

**>2077A.14.11**

**CTTCAAGAGTGATGGGCACAACCGGAGATACCCAGTCCACGGGCCCGTGGATGCTAAAAC**

**>2077A.14.12**

**CTTCAAGAGTGACAGGCACAACCGGAGATACCCAGTCCACGGGCCCGTGGACGCTAAAAC**

**>2077B.37G.Denmark**

**CTTCAAGAGTGACAGGCACAACCGGAGATACCCAGTCCACGGGCCCGTGGACGCTAAAAC**

**>2077B.38G.Denmark**

**CTTCAAGAGTGATGGGCACAACCGGAGATACCCAGTCCACGGGCCCGTGGATGCTAAAAC**

**>2077B.44G.Denmark**

**CTTCAAGAGTGATGGGTACAACCGGAGATACTCAGTCCACGGGCCCGTGGATGCTAAAAC**

**>2077B.45G.Denmark**

**CTTCAAGAGTGATGGGCACAACCGGAGATACCCAGTCCACGGGCCCGTGGATGCTAAAAC**

**>2077B.46G.Denmark**

**CTTCAAGAGTGATGGGCACAACCGGAGATACCCAGTCCACGGGCCCGTGGATGCTAAAAC**

**>2077.C..Denmark_1-RAG**

**CTTCAAGAGTGATGGGCACAACCGGAGATACCCAGTCCACGGGTCCGTGGATGCTAAAAC**

**>2077.C..Denmark_1-RAG**

**CTTCAAGAGTGATGGGCACAACCGGARATACCCAGTCCACGGGCCYGTGGATGCTAAAAC**

**>2077.C..Denmark_1-RAG**

**CTTCAAGAGCGATGGGCACAACCGGAGATACCCAGTCCACGGGCCCGTGGATGCTAAAAC**

**>2077.C.Denmark_1-RAG**

**CTTCAAAAGTGATGGGCACAACCGGAGATACCCAGTCCACGGGCCCGTGGATGCTAAAAC**

**>2077.C.Denmark_1-RAG**

**CTTCAAGAGTGATGGGCACAACCGGARATGCCCAGTCCACGGGYCCGTGGATGCTAAAAC**

**>2077.C.Denmark_1-RAG**

**CTTCAAGAGTGATGGGCACAACCGGAAATACCCAGTCCACGGGCCCGTGGATGCTAAAAC**

**>2077.C.Denmark_1-RAG**

**CTTCAAGAGTGATGGGCACAACCGGAGATACCCAGTCCACGGGTCCGTGGATGCTAAAAC**

**>**

**>18841A.1.1**

**CTTCAAGAGTGATGGGCACAACCGGAGATACCCAGTCCACGGGTCCGTGGATGCTAAAAC**

**>18841A.1.2**

**CTTCAAGAGTGATGGGCACAACCGGAGATACCCAGTCCATGGGCCCGTGGATGCTAAAAC**

**>18841A.1.3**

**CTTCAAGAGTGATGAGCACAACCGGAGATACCCAGTCCATGGGCCCGTGGATGCTAAAAC**

**>18841B.2.4**

**CTTCAAGAGTGACGGGCACAACCGGAGATACCCAGTCCACGGGCCCGTGGATGCTAAAAC**

**>18841B.2.5**

**CTTCAAGAGTGATGGGCATAACCGGAGATACCCAGTCCATGGGCCCGTGGATGCTAAAAC**

**>18841B.2.6**

**CTTCAAGAGTGATGGGCACGACCGGAGATACCCAGTCCACGGGCCCGTGGATGCTAAAAC**

**>18841C.8.1**

**CTTCAAGAGTGATGGGCACAACCGAAGATACCCAGTCCACGGGCCCGTGGATGCTAAAAC**

**>18841C.8.2**

**CTTCAAGAGTGATGGGCACAACCGGAGATACCCAGTCCACGGGCCCGTGGATGCTAAAAC**

**>18841C.8.3**

**CTTCAAGAGTGATGGGCACAACCGGAGATACCCAGTCCACGGGCCCGTGGATGCTAAAAC**

**>**

**>18843A.5.10**

**CTTCAAGAGTGGTGGGCACAACCGGAGATACCCAGTCCACGGGCCTGTGGATGCTAAAAC**

**>18843A.5.11**

**CTTCAAGAGTGATGGGCACAACCGGAGATACCCAGTCCACGGGCCCGTGGATGCTAAAAC**

**>18843A.5.1**

**CTTCAAGAGTGATGGGCACAACCGGAGATACCCAGTCCACGGGCCCGTGGATGCTAAAAC**

**>18843A.5.2**

**CTTCAAGTGTGATGGGCACAACCGGAGATACCCAGTCCACGGGCCCGTGGATGCTAAAAC**

**>18843A.5.3**

**CTTCAAGAGTGATGGGCACAACCGGAGATACCCAGTCCACGGGCCCGTGGATGCTAAAAC**

**>18843B.6.4**

**CTTCAAGAGTGATGGGCACAACCGGAGATACCCAGTCCACGGGCCCGTGGATGCTAAAAC**

**>18843B.6.5**

**CTTCAAGAGTGATGGGCACAACCGGAGATACCCAGTCCACGGGCCCGTGGATGCTAAAAC**

**>**

**>18844A.7.7**

**CTTCAAGAGTGATGGGCACAACCGGAGATACCCAGTCCACGGGCCCGTGGATGCTAAAAC**

**>18844A.7.8**

**CTTCAAGAGTGATGGGCACAACCGGAGATACCCAGTCCACGGGCCCGTGGATGCTAAAAC**

**>18844A.7.9**

**CTTCAAGAGTGATGGGCACAACCGGAGATACCCAGTCCACGGGCCCGTGGATGCTAAAAC**

**>18844B.1.4**

**CTTCAAGAGTGATGGGCACAACCGGAGATACCCAGTCCACGGGCCCGTGGATGCTAAAAC**

**>18844B.1.5**

**CTTCAAGAGTGATGGGCACAACTGGAGATACCCAGTCCACGGGCCCGTGGATGCTAAAAC**

**>18844B.1.6**

**CTTCAAGAGTGATGGGCACAACCGGAGATACCCAGTCCACGGGCCCGTGGATGCTAAAAC**

**>**

**>18845A.4.7**

**CTTCAAGAGTGATGGGCACAACCGGAGATACCCAGTCCACGGGCCCGTGGATGCTAAAAC**

**>18845A.4.8**

**CTTCAAGAGTGATGGGCACAACCGGAGATACCCAGTCCACGGGCCCGTGGATGTTAAAAC**

**>18845A.4.9**

**CTTCAAGAGTGATGGGCACAACCGGAGATACCCAGTCCATGGGCCCGTGGATGCTAAAAC**

**>18845B.13**

**CTTCAAGAGTGATGGGCACAACCGGAGATACCCAGTCCACGGGCCCGTGGATGCTAAAAC**

**>18845B.14**

**CTTCAAGAGTGACAGGCACAACCGGAGATACCCAGTCCACGGGCCCGCGGACGCTAAAAC**

**>**

**>18846A.4.8**

**CTTCAAGAGTGACGGGCACAACCGGAGATACCCAGTCCACGGGCCCGTGGATGCTAAAAC**

**>18846A.4.9**

**CTTCAAGAGTGATGGGCACGACCGGAGATACCCAGTCCACGGGCCCGTGGATGCTAAAAC**

**>18846A.4.10**

**CTTCAAGAGTGATGGGCACAACCGGAGATACCCAGTCCACGGGCCCGTGGATGCTAAAAC**

**>18846B.9.9**

**CTTCAAGAGTGATGGGCATAACCGGAGATACCCAGTCCACGGGCCCGTGGATGCTAAAAC**

**>18846B.9.10**

**CTTCAAGAGTGATGAGCACAACCGGAGATACCCAGTCCATGGGCCCGTGGATGCTAAAAC**

**>18846.C.Denmark_1-RAG**

**CTTCAAGAGTGGTGGGCACAACCGGAGATACCCAGTCCACGGGCCCGTGGATGCTAAAAC**

**>18846.C.Denmark_1-RAG**

**CTTCAAGAGTGGTGGGCACAACCGGAGATACCCAGTCCACGGGCCCGTGGATGCTAAAAC**

**>18846.C.Denmark_1-RAG**

**CTTCAAGAGTGGTGGGCACAACCGGAGATACCCAGTCCACGGGCCCGTGGATGCTAAAAC**

**>18846.C.Denmark_1-RAG**

**CTTCAAGAGTGATGGGCACAACCGGAGATACCCAGTCCGCGGGCCCGTGGATGCTAAAAC**

**>18846.C.Denmark_1-RAG**

**CTTCAAGAGTGATGGGCACAACCGGAGATACCCAGTCCACGGGCCCGTGGATGCTAAAAC**

**>**

**>1899.8.6.29.A.Denmark**

**TTTCAAGAGTGACGGGCACAACCGGAGATACCCAGTCCATGGGCCCGTGGACGCTAAAAC**

**>1899.8.6.29.A.Denmark**

**TTTCAAGAGTGACGGGCACAACCGGAGATACCCAGTCCATGGGCCCGTGGACGCTAAAAC**

**>1899.8.6.29.A.Denmark**

**TTTCAAGAGTGACGGGCACAACCGGAGATACCCAGTCCATGGGCCCGTGGACGCTAAAAC**

**>1899.8.6.29.A.Denmark**

**TTTCAAGAGTGACGGGCACAACCGGAGATACCCAGTCCATGGGCCCGTGGACGCTAAAAC**

**>1899.8.6.29.A.Denmark**

**TTTCAAGAGTGACGGGCACAACCGGAGATACCCAGTCCATGGGCCCGTGGACGCTAAAAC**

**>1899.8.6.29.A.Denmark**

**TTTCAAGAGTGACGGGCACAACCGGAGATACCCAGTCCATGGGCCCGTGGACGCTAAAAC**

**>1899.8.6.29.A.Denmark**

**TTTCAAGAGTGACGGGCACAACCGGAGATACCCAGTCCATGGGCCCGTGGACGCTAAAAC**

**>1899.8.6.29.A.Denmark**

**TTTCAAGAGTGACGGGCACAACCGGAGATACCCAGTCCATGGGCCCGTGGACGCTAAAAC**

**>1899.8.6.29.A.Denmark**

**TTTCAAGAGTGACGGGCACAACCGGAGATACCCAGTCCATGGGCCCGTGGACGCTAAAAC**

**>1899.8.6.29.A.Denmark**

**TTTCAAGAGTGACGGGCACAACCGGAGATACCCAGTCCATGGGCCCGTGGACGCTAAAAC**

**>1899.8.6.29.A.Denmark**

**TTTCAAGAGTGACGGGCACAACCGGAGATACCCAGTCCATGGGCCCGTGGACGCTAAAAC**

**>1899.8.6.29.A.Denmark**

**TTTCAAGAGTGACGGGCACAACCGGAGATACCCAGTCCATGGGCCCGTGGACGCTAAAAC**

**>1899.8.6.29.A.Denmark**

**TTTCAAGAGTGACGGGCACAACCGGAGATACCCAGTCCATGGGCCCGTGGACGCTAAAAC**

**>**

**>1899.8.6.28.A.Denmark**

**TTTCAAGAGTGACGGGCACAACCRGAGATACCCAGTCCATGGGCCCGNGGACGCTAAAAC**

**>1899.8.6.28.A.Denmark**

**TTTCAAGAGTGACGGGCACAACCGGAGATACCCAGTCCATGGGCCCGTGGACGCTAAAAC**

**>1899.8.6.28.A.Denmark**

**TTTCAAGAGTGACGGGCACAACCGGAGATACCCAGTCCATGGGCCCGTGGACGCTAAAAC**

**>1899.8.6.28.A.Denmark**

**TTTCAAGAGTGACGGGCACAACCGGAGATACCCAGTCCATGGGCCCGTGGACGCTAAAAC**

**>1899.8.6.28.A.Denmark**

**TTTCAAGAGTGACGGGCACAACCGGAGATACCCAGTCCATGGGCCCGTGGACGCTAAAAC**

**>1899.8.6.28.A.Denmark**

**TTTCAAGAGTGACGGGCACAACCGGAGATACCCAGTCCATGGGCCCATGGACGCTAAAAC**

**>1899.8.6.28.A.Denmark**

**TTTCAAGAGTGACGGGCACAACCRGAGATACCCAGTCCATGGGCCCGTGGACGCTAAAAC**

**>1899.8.6.28.A.Denmark**

**TTTCAAGAGTGACGGGCACAACCGGAGATACCCAGTCCATGGGCCCGTGGACGCTAAAAC**

**>1899.8.6.28.A.Denmark**

**TTTCAAGAGTGACGGGCACAACCGGAGATACCCAGTCCATGGGCCCGTGGACGCTAAAAC**

**>**

**>1888.7.9.5.A.Denmark**

**TTTCAAGAGTGACGGGCACAACCGGAGATACCCAGTCCATGGGCCCGTGGACGCTAAAAC**

**>1888.7.9.5.A.Denmark**

**TTTCAAGAGTGACGGGCACAACCGGAGATACCCAGTCCATAGGCCCGTGGACGCTAAAAC**

**>1888.7.9.5.A.Denmark**

**TTTCAAGAGTGACGGGCACAACCGGAGATACCCAGTCCATGGGCCCGTGGACGCTAAAAC**

**>1888.7.9.5.A.Denmark**

**TTTCAAGAGTGACGGGCACAACCGGAGATACCCAGTCCATGGGCCCGTGGACGCTAAAAC**

**>1888.7.9.5.A.Denmark**

**TTTCAAGAGTGACGGGCACAACCGGAGATACCCAGTCCATGGGCCCGTGGACGCTAAAAC**

**>1888.7.9.5.A.Denmark**

**TTTCAAGAGTGACGGGCACAACCGGAGATACCCAGTCCATGGGCCCGTGGACGCTAAAAC**

**>1888.7.9.5.A.Denmark**

**TTTCAAGAGTGACGGGCACAACCGGAGATACCCAGTCCATGGGCCCGTGGACGCTAAAAC**

**>1888.7.9.5.A.Denmark**

**TTTCAAGAGTGACGGGCACAACCGGAGATACCCAGTCCATGGGCCCGTGGACGCTAAAAC**

**>1888.7.9.5.A.Denmark**

**TTTCAAGAGTGACGGGCACAACCGGAGATACCCAGTCCATAGGCCCGTGGACGCTAAAAC**

**>1888.7.9.5.A.Denmark**

**TTTCAAGAGTGACGGGCACAACCGGAGATACCCAGTCCATAGGCCCGTGGACGCTAAAAC**

**>1888.7.9.5.A.Denmark**

**TTTCAAGAGTGACGGGCACAACCGGAGATACCCAGTCCATAGGCCCGTGGACGCTAAAAC**

**>1888.7.9.5.A.Denmark**

**TTTCAAGAGTGACGGGCACAACCGGAGATACCCAGTCCATAGGCCCGTGGACGCTAAAAC**

**>1888.7.9.5.A.Denmark**

**TTTCNNGANTGACGGGCACAACCGGAGATACCCAGTCCATGGGCCCGTGGACGCTAAAAC**

**>1888.7.9.5.A.Denmark**

**TTTCAAGAGTGACGGGCACAACCGGAGATACCCAGTCCATAGGCCCGTGGACGCTAAAAC**

**>1888.7.9.5.A.Denmark**

**TTTCAAGAGTGACGGGCACAACCGGAGATACCCAGTCCATGGGCCCGTGGACGCTAAAAC**

**>**

**>RAG B**

**>18841A.1.2**

**TGCATTCTC-CGGTGCCTCAAGGTCATGGGCAGCTATTGTCCCTCTTGCCGATATCCATG**

**CTTCCCTATTGACCTGGAGAGTCC**

**>18841A.1.3**

**TGCATTTTC-CGGTGCCTCAAGGTCATGGGCAGCTATTGTCCCTCTTGCCGATATCCATG**

**CTTCCCTACTGACCTGGAGAGTCC**

**>18841B.2.4**

**TGCATTCTC-CGGTGCCTCAAGGTCATGGGCAGCTATTGTCCCTCTTGCCGATATCCATG**

**CTTCCCTACTGACCTGGAGAGTCC**

**>18841B.2.5**

**TGCATTCTC-CGGTGCCTCAAGGTCATGGGCAGCTATTGTCCCTCTTGCCGATATCCATG**

**CTTCCCTACTGACCTGGAGAGTCC**

**>18841B.2.6**

**TGCATTCTC-CGGTGCCTCAAGGTCATGGGCAGCTATTGTCCCTCTTGCCGATATCCATG**

**CTTCCCTACTAACCTGGAGAGTCC**

**>**

**>18845A.1.2**

**TGCATTCTC-CGGTGCCTCAAGGTCATGGGCAGCTATTGTCCCTCTTGCCGATATCCATG**

**CTTCCCTACTGACCTGGAGAGTCC**

**>18845A.1.3**

**TGCATTCTC-CGGTGCCTCAAGGTCATGGGCAGCTATTGTCCCTCTTGCCGATATCCATG**

**CTTCCCTACTGACCTGGAGAGTCC**

**>18845B.2.4**

**TGCATTCTC-CGGTGCCTCAAGGTCATGGGCAGCTATTGTCCCTCTTGCCGATATCCATG**

**CTTCCCTACTGACCTGGAGAGTCC**

**>18845B.2.5**

**TGCATTCTC-CGGTGCCTCAAGGTCATGGGCAGCTATTGTcCCTCTTGCCGATATCCATG**

**CTTCCCTACTGACCTGGAGAGTCC**

**>**

**>18846A.14.16**

**TGCATTCTCTTGGTGCCTCAAGGTCATGGGCAGCTATTGTCCCTCTTGCCGATATCCATG**

**CTTCCCTACTGACCTGGAGAGTCC**

**>18846A.14.17**

**TGCATTCTCTCGGTGCCTCAAGGTCATGGGCAGCTATTGTCCCTCTTGCCGATATCCATG**

**CTTCCCTACTGACCTGGAGAGTCC**

**>18846B.15.18**

**TGCATTCTCTCGGTGCCTCAAGGTCATGGGCAGCTATTGTCCCTCTTGCCGATATCCATG**

**CTTCCCTACTGACCTGGAGAGTCC**

**>18846B.15.19**

**TGCATTCTCTCGGTGCCTCAAGGTCATGGGCAGCTATTGTCCCTCTTGCCGATATCCATG**

**CTTCCCTACTGACCTGGAGAGTCC**

**>**

**>2078A.1.2**

**TGCATTCTC-CGGTGCCTCAAGGTCATGGGCAGCTACTGTCCCTCGTGCCGATATCCATG**

**CTTCCCTACTGACCTGGAGAGTCC**

**>2078A.1.3**

**TGCATTCTC-CGGTGCCTCAAGGTCATGGGCAGCTACTGTCCCTCGTGCCGATATCCATG**

**CTTCCCTACTGACCTGGAGAGTCC**

**>2078B.2.4**

**TGCATTCTC-CGGTGCCTCAAGGTCATGGGCAGCTACTGTCCCTCGTGCCGATATCCATG**

**CTTCCCTACTGACCTGGAGAGTCC**

**>2078B.2.5**

**TGCATTCTC-CGGTGCCTCAAGGTCATGGGCAGCTACTGTCCCTCGTGCCGATATCCATG**

**CTTCCCTACTGACCTGGAGAGTCC**

**>2078B.2.6**

**TGCATTCTC-CGGTGCCTCAAGGTCATGGGCAGCTACTGTCCCTCGTGCCGATATCCATG**

**CTTCCCTACTGACCTGGAGAGTCC**

**>**

**>2079A.D1**

**TGCATTCTC-CGGTGCCTCAAGGTCATGGGCAGCTACTGTCCCTCGTGCCGATATCCATG**

**CTTCCCTACTGACCTGGAGAGTCC**

**>2079A.D3**

**TGCATTCTC-CGGTGCCTCAAGGTCATGGGCAGCTACTGTCCCTCGTGCCGATATCCATG**

**CTTCCCTACTGACCTGGAGAGTCC**

**>2079A.D4**

**TGCATTCTC-CGGTGCCTCAAGGTCATGGGCAGCTACTGTCCCTCGTGCCGATATCCATG**

**CTTCCCTACTGACCTGGAGAGTCC**

**>2079A.D5**

**TGCATTCTC-CGGTGCCTCAAGGTCATGGGCAGCTACTGTCCCTCGTGCCGATATCCATG**

**CTTCCCTACTGACCTGGAGAGTCC**

**>2079B.U1**

**TGCATTCTC-CGGTGCCTCAAGGTCATGGGCAGCTACTGTCCCTCGTGCCGATATCCATG**

**CTTCCCTACTGACCTGGAGAGTCC**

**>2079B.U4**

**TGCATTCTC-CGGTGCCTCAAGGTCATGGGCAGCTACTGTCCCTCGTGCCGATATCCATG**

**CTTCCCTACTGACCTGGAGAGTCC**

**>**

**>18606A.4.4**

**TGCATTCTC-CGGTGCCTCAAGGTCATGGGCAGCTACTGTCCCTCGTGCCGATATCCATG**

**CTTCCCTACTGACCTGGAGAGTCC**

**>18606A.4.5**

**TGCATTCTC-CGGTGCCTCAAGGTCATGGGCAGCTACTGTCCCTCGTGCCGATATCCATG**

**CTTCCCTACTGACCTGGAGAGTCC**

**>18606A.4.6**

**TGCATTCTC-CGGTGCCTCAAGGTCATGGGCAGCTACTGTCCCTCGTGCCGATATCCATG**

**CTTCCCTACTGACCTGGAGAGTCC**

**>18606B.5.7**

**TGCATTCTC-CGGTGCCTCAAGGTCATGGGCAGCTACTGTCCCTCGTGCCGATATCCATG**

**CTTCCCTACTGACCTGGAGAGTCC**

**>18606B.5.8**

**TGCATTCTC-CGGTGCCTCAAGGTCATGGGCAGCTACTGTCCCTCGTGCCGATATCCATG**

**CTTCCCTACTGACCTGGAGAGTCC**

**>18606B.5.9**

**TACATTCTC-CGGTGCCTCAAGGTCATGGGCAGCTACTGTCCCTCGTGCCGATATCCATG**

**CTTCCCTACTGACCTGGAGAGTCC**

**>**

**>18607A.1.6**

**TGCATTCTC-CGATGCCTCAAGGTCATGGGCAGCTACTGTCCCTCGTGCCGATATCCATG**

**CTTCCCTACTGACCTAGAGAGTCC**

**>18607A.1.7**

**TGCATTCTC-CGATGCCTCAAGGTCATGGGCAGCTACTGTCCCTCGTGCCGATATCCATG**

**CTTCCCTACTGACCTAGAGAGTCC**

**>18607B.2.8**

**TGCATTCTC-CGATGCCTCAAGGTCATGGGCAGCTACTGTCCCTCGTGCCGATATTCATG**

**CTTCCCTACTGACCTAGAGAGTCC**

**>18607B.2.9**

**TGCATTCTC-CGATGCCTCAAGGTCATGGGCAGCTACTGTCCCTCGTGCCGATATCCATG**

**CTTCCCTACTGACCTAGAGAGTCC**

**>**

**>2074A.2.1**

**TGCATTCTC-CGGTGCCTCAAGGTCATGGGCAGCTACTGTCCCTCGTGCCGATATCCATG**

**CTTCCCTACTGACCTGGAGAGTCC**

**>2074A.2.2**

**TGCATTCTC-CGGTGCCTCAAGGTCATGGGCAGCTACTGTCCCTCGTGCCGATATCCATG**

**CTTCCCTACTGACCTGGAGAGTCC**

**>2074A.2.3**

**TGCATTCTC-CGGTGCCTCAAGGTCATGGGCAGCTACTGTCCCTCGTGCCGATATCCATG**

**CTTCCCTACTGACCTGGAGAGTCC**

**>2074B.12.10**

**TGCATTCTC-CGGTGCCTCAAGGTCATGGGCAGCTACTCTCCCTCGTGCCGATATCCATG**

**CTTCCCTACTGACCTGGAGAGTCC**

**>2074B.12.15**

**TGCATTCTC-CGGTGCCTCAAGGTCATGGGCAGCTACTGTCCCTCGTGCCGATATCCATG**

**CTTCCCTACTGACCTGGAGAGTCC**

**>**

**>2075A.1**

**TGCATTCTC-CGGTGCCTCAAGGTCATGGGCAGCTACTGTCCCTCGTGCCGATATCCATG**

**CTTCCCTACTGACCTGGAGAGTCC**

**>2075A.2**

**TGCATTCTC-CGGTGCCTCAAGGACATGGGCAGCTACTGTCCCTCGTGCCGATATCCATG**

**CTTCCCTACTGACCTGGAGAGTCC**

**>2075B.11**

**TGCATTCTC-CGGTGCCTCAAGGTCATGGGCAGCTACTGTCCCTCGTGCCGATATCCATG**

**CTTCCCTACTGACCTGGAGAGTCC**

**>2075B.12**

**TGCATTCTC-CGGTGCCTCAAGGTCATGGGCAGCTACTGTCCCTCGTGCCGATATCCATG**

**CTTCCCTACTGACCTGGAGAGTCC**

**>**

**>GHR A**

**>18841A.4.7**

**CCCTCCATTACCCTGACAATGGAAGACAAACCACAGCCACTTCTGGGCAGTGAAACTGAG**

**TCAACCCACCAACTCGTCCCTACACCAATGAGCAATCCCGTGTCACTA**

**>18841A.4.8**

**CCCTCCATTACCCTGACAATGGAAGACAAACCACAGCCACTTCTGGGCAGTGAAACTGAG**

**TCAACCCACCAACTCGTCTCTACACCAATGAGCAATCCCGTGTCACTA**

**>18841B.5.10**

**CCCTCCATTACCCTGACAATGGAAGACAAACCACAGCCACTTCTGGGCAGTGAAACTGAG**

**TCAACCCACCAACTCGTCTCTACACCAATGAGCAATCCCGTGTCACTA**

**>18841B.5.11**

**CCCTCCATTACCCTGACAATGGAAGACAAACCACAGCCACTTCTGGGCAGTGAAACTGAG**

**TCAACCCACCAACTCGTCTCTACACCAATGAGCAATCCCGTGTCACTA**

**>18841B.15.12**

**CCCTCCATTACCCTGACAATGGAAGACAAACCACAGCCACTTCTGGGCAGTGAAACTGAG**

**TCAACCCACCAACTCGTCTCTACACCAATGAGCAATCCCGTGTCACTA**

**>**

**>18845A.4.8**

**CCCTCCATTACCCTGACAATGGAAGACAAACCACAGCCACTTCTGGGCAGTGAAACTGAG**

**TCAACCCACCAACTCGTCTCTACACCAATGAGCAATCCCGTGTCACTA**

**>18845A.4.9**

**CCCTCCATTACCCTGACAATGGAAGACAAACCACAGCCACTTCTGGGCAGTGAAACTGAG**

**TCAACCCACCAACTCGTCTCTACACCAATGAGCAATCCCGTGTCACTA**

**>18845B.5.10**

**CCCTCCATTACCCTGACAATGGAAGACAAACCACAGCCACTTCTGGGCAGTGAAACTGAG**

**TCAACCCACCAACTCGTCTCTACACCAATGAGCAATCCCGTGTCACTA**

**>18845B.5.12**

**CCCTCCATTACCCTGACAATGGAAGACAAACCACAGCCACTTCTGGGCAGTGAAACTGAG**

**TCAACCCACCAACTCGTCTCTACACCAATGAGCAATCCCGTGTCACTA**

**>**

**>18846A.7.1**

**CCCTCCATTACCCTGACAATGGAAGACAAACCACAGCCACTTCTGGGCAGTGAAACTGAG**

**TCAACCCACCAACTCGTCTCTACACCAATGAGCAATCCCGTGTCACTA**

**>18846A.7.2**

**CCCTCCATTACCCTGACAATGGAAGACAAACCACAGCCACCTCTGGGCAGTGAAACTGAG**

**TCAACCCACCAACTCGTCTCTACACCAATGAGCAATCCCGTGTCACTA**

**>18846A.7.3**

**CCCTCCATTACCCTGACAATGGAAGACAAACCACAGCCACTTCTGGGCAGTGAAACTGAG**

**TCAACCCACCAACTCGTCTCTACACCAATGAGCAATCCCGTGTCACTA**

**>18846B.8.4**

**CCCTCCATTACCCTGACAATGGAAGACAAACCACAGCCACTTCTGGGCAGTGAAACTGAG**

**TCAACCCACCAACTCGTCTCTACACCAATGAGCAATCCCGTGTCACTA**

**>18846B.8.6**

**CCCTCCATTACCCTGACAATGGAAGACAAACCACAGCCACTTCTGGGCAGTGAAACTGAG**

**TCAACCCACCAACTCGTCTCTACACCAATGAGCAATCCCGGGTCACTA**

**>**

**>2078A.4.8**

**CCCTCCATTACCCTGACAATGGAAGACAAACCACAGCCACTTCTGGGCAGTGAAACTGAA**

**TCAACCCACCAACTCGTCTCTACACCAATGAGCAATCCCGTGTCACTG**

**>2078A.4.9**

**CCCTCCATTACCCTGACAATGGAAGACAAACCACAGCCACTTCTGGGCAGTGAAACTGAA**

**TCAACCCACCAACTCGTCTCTACACCAATGAGCAATCCCGTGTCACTG**

**>2078B.5.10**

**CCCTCCATTACCCTGACAATGGAAGACAAACCACAGCCACTTCTGGGCAGTGAAACTGAA**

**TCAACCCACCAACTCGTCTCTACACCAATGAGCAATCCCGTGTCACTG**

**>2078B.5.11**

**CCCTCCATTACCCTGACAATGGAAGACAAACCACAGCCACTTCTGGGCAGTGAAACTGAA**

**TCAACCCACCAACTCGTCTCTACACCAATGAGCAATCCCGTGTCACTG**

**>2078B.5.12**

**TCCTCCATTACCCTGACAATGGAAGACAAACCACAGCCACTTCTGGGCAGTGAAACTGAA**

**TCAACCCACCAACTCGTCTCTACACCAATGAGCAATCCCGTGTCACTG**

**>**

**>2079A.D6**

**CCCTCCATTACCCTGACAATGGAAGACAAACCACAGCCACTTCTGGGCAGTGAAACTGAA**

**TCAACCCACCAACTCGTCTCTACACCAATGAGCAATCCCGTGTCACTG**

**>2079A.D8**

**CCCTCCATTACCCTGACAATGGAAGACAAACCACAGCCACTTCTGGGCAGTGAAACTGAA**

**TCAACCCACCAACTCGTCTCTACACCAATGAGCAATCCCGTGTCACTG**

**>2079A.D10**

**CCCTCCATTACCCTGACAATGGAAGACAAACCACAGCCACTTCTGGGCAGTGAAACTGAA**

**TCAACCCACCAACTCGTCTCTACACCAATGAGCAATCCCGTGTCACCG**

**>2079B.U1**

**CCCTCCATTACCCTGACAATGGAAGACAAACCACAGCCACTTCTGGGCAGTGAAACTGAA**

**TCAACCCATCAACTCGTCTCTACACCAATGAGCAATCCCGTGTCACTG**

**>2079B.U2**

**CCCTCCATTACCCTGACAATGGAAGACAAACCACAGCCACTTCTGGGCAGTGAAACTGAA**

**TCAACCCACCAACTCGTCTCTACACCAATGAGCAATCCCGTGTCACTG**

**>2079B.U3**

**CCCTCCATTACCCTGACAATGGAAGACAAACCACAGCCACTTCTGGGCAGTGAAACTGAA**

**TCAACCCACCAACTCGTCTCTACACCAATGAGCAATCCCGTGTCACTG**

**>**

**>18606A.7.12**

**CCCTCCATTACCCTGACAATGGAAGACAAACCACAGCCACTTCTGGGCAGTGAAACTGAA**

**TCAACCCACCAACTCGTCTCTACACCAATGAGCAATCCCGTGTCACTG**

**>18606B.8.13**

**CCCTCCATTACCCTGACAATGGAAGACAAACCACAGCCACTTCTGGGCAGTGAAACTGAA**

**TCAACCCACCAACTCGTCTCTACACCAATGAGCAATCCCGTGTCACTG**

**>18606C.7.5**

**CCCTCCATTACCCTGACAATGGAAGACAAACCACAGCCACTTCTGGGCAGTGAAACTGAA**

**TCAACCCACCAACTCGTCTCTACACCAATGAGCAATCCTGTGTCACTG**

**>18606C.7.6**

**CCCTCCATTACCCTGACAATGGAAGACAAACCACAGCCATTTCTGGGCAGTGAAATTGAA**

**TCAACCCACCAACTCGTCTCTACACCAATGAGCAATCCCGTGTCACTG**

**>18606D.8.8**

**CCCTCCATTACCCTGACAATGGAAGACAAACCACAGCCACTTCTGGGCAGTGAAACTGAA**

**TCAACCCACCAACTCGTCTCTACACCAATGAGCAATCCCGTGTCACTG**

**>18606D.8.9**

**CCCTCCATTATCCTGACAATGGAAGACAAACCACAGCCACTTCTGGGCAGTGAAACTGAA**

**TCAACCCACCAACTCGTCTCTACACCAATGAGCAATCCCGTGTCACTG**

**>**

**>18607A.2.1**

**CTCTCCATTACCCTGACAATGGAAGACAAACCACAGCCACTTCTGGGCAGTGAAACTGAA**

**TCAACCCACCAACTCGTCTCTACACCAATGAGCAATCCTGTGTCACTG**

**>18607A.2.2**

**CCCTCCATTACCCTGACAATGGAAGACAAACCACAGCCACTTCTGGGCAGTGAAACTGAA**

**TCAACCCACCAACTCGTCTCTACACCAATGAGCAATCCCGTGTCACTG**

**>18607B.1**

**CCCTCCATTACCCTGACAATGGAAGACAAACCACAGCCACTTCTGGGCAGTGAAACTGAA**

**TCAACCCACCAACTCGTCTCTACACCAATGAGCAATCCCGTGTCACTG**

**>18607B.2**

**CCCTCCATTACCCTGACAATGGAAGACAAACCACAGCCACTTCTGGGCAGTGAAACTGAA**

**TCAACCCACCAACTCGTCTCTACACCAATGAGCAATCCCGTGTCACTG**

**>18607B.3**

**CCCTCCATTACCCTGACAATGGAAGACAAACCACAGCCACTTCTGGGCAGTGAAACTGAA**

**TCAACCCACCAACTCGTCTCTACACCAATGAGCAATCCCGTGTCACTG**

**>**

**>2074A.5.9**

**CCCTCCATTACCCTGACAATGGAAGACAAACCACAGCCACTTCTGGGCAGTGAAACTGAA**

**TCAACCCACCAACTCGTCTCTACACCAATGAGCAATCCCGTGTCACTG**

**>2074B.4.4**

**CCCTCCATTACCCTGACAATGGAAGACAAACCACAGCCACTTCTGGGCAGTGAAACTGAA**

**TCAACCCACCAACTCGTCTCTACACCAATGAGCAATCCCGTGTCACTG**

**>2074B.4.6**

**CCCTCCATTACCCTGACAATGGAAGACAAACCACAGCCACTTCTGGGCAGTGAAACTGAA**

**TCAACCCACCAACTCGTCTCTACACCAATGAGCAATCCCGTGTCACTG**

**>2074C.5.19**

**CCCTCCATTACCCTGACAATGGAAGACAAACCACAGCCACTTCTGGGCAGTGAAACTGAA**

**TCAACCCACCAACTCGTCTCTACACCAATGAGCAATCCCGTGTCACTG**

**>2074C.5.20**

**CCCTCCATTACCCTGACAATGGAAGACAAACCACGGCCACTTCTGGGCAGTGAAACTGAA**

**TCAACCCACCAACTCGTCTCTACACCAATGAGCAATCCCGTGTCACTG**

**>**

**>2075A.1.1**

**CCCTCCATTACCCTGACAATGGAAGACAAACCACAGCCACTTCTGTGCAGTGAAACTGAA**

**TCAACCCACCAACTCGTCTCTACACCAATGAGCAATCCCGTGTCACTG**

**>2075A.1.2**

**CCCTCCATTACCCTGACAATGGAAGACAAACCACAGCCACTTCTGGGCAGTGAAACTGAA**

**TCAACCCACCAACTCGTCTCTACACCAATGAGCAATCCCGTGTCACTG**

**>2075B.2.1**

**CCCTCCATTACCCTGACAATGGAAGACAAACCACAGCCACTTCTGGGCAGTGAAACTGAA**

**TCAACCCACCAACTCGTCTCTACACCAATGAGCAATCCCGTGTCACTG**

**>2075B.2.2**

**CCCTCCATTACCCTGACAATGGAAGACAAACCACAGCCACTTCTGGGCAGTGAAACTGAA**

**TCAACCCACCAACTCGTCTCTACACCAATGAGCAATCCCGTGTCACTG**

**>**

**>GHR B**

**>18841A.7.13**

**CCGCTCCAGATGCTGAGATGCCTGTCCCAGACTACACCACGGTTCACACTGTGCAGTCTC**

**CAAGGGGCCT**

**>18841A.7.14**

**CCGCTCCAGATGCTGAGATGCCTGTCCCAGACTACACCACGGTTCACACTGTGCAGTCTC**

**CAAGGGGCCT**

**>18841A.7.15**

**CCGCTCCAGATGCTGAGATGCCTGTCCCAGACTACACCACGGTTCACACTGTGCAGTCTC**

**CAAGGGGCCT**

**>18841B.8.17**

**CCGCTCCAGATGCTGAGATGCCTGTCCCAGACTACACCACGGTCCACACTGTGCAGTCTC**

**CAAGGGGCCT**

**>18841B.8.18**

**CCGCTCCAGATGCTGAGATGCCTGTCCCAGACTACACCACGGTTCACACTGTGCAGTCTC**

**CAAGGGGCCT**

**>**

**>18845A.7.13**

**CCGCTCCAGATGCTGAGATTCCCGTCCCAGACTACACCACGGTTCACACTGTGCAGTCTC**

**CAAGGGGCCT**

**>18845A.7.14**

**CCGCTCCAGATGCTGAGATGCCTGTCCCAGACTACACCACGGTTCACACTGTGCAGTCTC**

**CAAGGGGCCT**

**>18845A.7.15**

**CCGCTCCAGATGCTGAGATGCCTGTCCCGGACTACACCACGGTTCACACTGTGCAGTCTC**

**CAAGGGGCCT**

**>18845B.8.16**

**CCGCTCCAGATGCTGAGATGCCTGTCCCAGACTACACCACGGTTCACACTGTGCAGTCTC**

**CAAGGGGCCT**

**>18845B.8.17**

**CCGCTCCAGATGCTGAGATGCCTGTCCCAGACTACACCACGGTTCACACTGTGCAGTCTC**

**CAAGGGGCCT**

**>18845B.8.18**

**CCGCTCCAGATGCTGAGATGCCCGTCCCAGACTACACCACGGTTCACACTGTGCAGTCTC**

**CAAGGGGCCT**

**>**

**>18846A.10.7**

**CCGCTCCAGATGCTGAGATGCCTGTCCCAGACTACACCACGGTTCACACTGTGCAGTCTC**

**CAAGGGGCCT**

**>18846A.10.8**

**CCGCTCCAGATGCTGAGATGCCTGTCCCAGACTACACCACGGTTCACACTGTGCAGTCTC**

**CAAGGGGCCT**

**>18846A.10.9**

**CCGCTCCAGATGCTGAGATGCCTGTCCCAGACTACACCACGGTTCACACTGTGCAGTCTC**

**CAAGGGGCCT**

**>18846B.11.11**

**CCGCTCCAGATGCTGAGATGCCTGTCCCAGACTACACCACGGTTCACACTGTGCAGTCTC**

**CAAGGGGCCT**

**>18846B.11.12**

**CCGCTCCAGATGCTGAGATGCCTGTCCCAGACTACACCACGGTTCACACTGTGCAGTCTC**

**CAAGGGGCCT**

**>**

**>2078A.7.13**

**CCGCTCCAGATGCTGAGATGCCTGTCCCAGACTATACCACGGTTCACACCGTGCAGTCTC**

**CAAGGGGCCT**

**>2078A.7.14**

**CCGCTCCAGATGCTGAGATGCCTGTCCCAGACTATACCACGGTTCACACCGTGCAGTCTC**

**CAAGGGGCCT**

**>2078A.7.15**

**TCGCTCCAGATGCTGAGATGCCTGTCCCAGACTATACCACGGTTCACACCGTGCAGTCTC**

**CAAGGGGCCT**

**>2078B.8.16**

**CCGCTCCAGATGCTGAGATGCCTGTCCCAGACTATACCACGGTTCACACCGTGCAGTCTC**

**CAAGGGGCCT**

**>2078B.8.17**

**CCGCTCCAGATGCTGAGATGCCTGTCCCAGACTATACCACGGTTCACACCGTGCAGTCTC**

**CAAGGGGCCT**

**>2078B.8.18**

**CCGCTCCAGATGCTGAGATGCCCGTCCCAGACTATACCACGGTTCACACCGTGCAGTCTC**

**CAAGGGGCCT**

**>**

**>2079A.1.7**

**CCGCTCCAGATGCTGAGATGCCTGTCCCAGACTATACCACGGTTCACACCGTGCAGTCTC**

**CAAGGGGCCT**

**>2079A.1.8**

**CCGCTCCAGATGCTGAGATGCCTGTCCCAAACTATACCACGGTTCACACCGTGCAGTCTC**

**CAAGGGGCCT**

**>2079A.1.9**

**CCGCTCCAGATGCTGAGATGCCTGTCCCAGACTATACCACGGTTCACACCGTGCAGTCTC**

**CAAGGGGCCT**

**>2079B.2.10**

**CCGCTCCAGATGCTGAGATGCCTGTCCCAGACTATACCACGGTTCACACCGTGCAGTCTC**

**CAAGGGGCCT**

**>2079B.2.1**

**CCGCTCCAGATGCTGAGATGCCTGTCCCAGACTATACCACGGTTCACACCGTGCAGTCTC**

**CAAGGGGCCT**

**>2079B.2.2**

**CCGCTCCAGATGCTGAGATGCCTGTCCCAGACTATACCACGGTTCACACCGTGCAGTCTC**

**CAAGGGGCCT**

**>2079.C.Denmark**

**--------------------------------TATACCACGGTTCACACCGTGCAGTCTC**

**CAAGGGGCCT**

**>2079.C.Denmark**

**--------------------------------TATACCACGGTTCACACCGTGCAGTCTC**

**CAAGGGGCCT**

**>2079.C.Denmark**

**--------------------------------TATACCACGGTTCACACCGTGCAGTCTC**

**CAAGGGGCCT**

**>2079.C.Denmark**

**--------------------------------TATACCACGGTTCACACCGTGCAGTCTC**

**CAAGGGGCCT**

**>2079.C.Denmark**

**--------------------------------TATACCACGGTTCACACCGTGCAGTCTC**

**CAAGGGGCCT**

**>2079.C.Denmark**

**--------------------------------TATACCACGGTTCACACCGTGCAGTCTC**

**CAAGGGGCCT**

**>2079.C.Denmark**

**--------------------------------TATACCACGGTTCACACCGTGCAGTCTC**

**CAAGGGGCCT**

**>2079.C.Denmark**

**--------------------------------TATACCACGGTTCACACCGTGCAGTCTC**

**CAAGGGGCCT**

**>2079.C.Denmark**

**--------------------------------TATACCACGGTTCACACCGTGCAGTCTC**

**CAAGGGGCCT**

**>2079.C.Denmark**

**--------------------------------TATACCACGGTTCACACCGTGCAGTCTC**

**CAAGGGGCCT**

**>2079.C.Denmark**

**--------------------------------TATACCACGGTTCACACCGTGCAGTCTC**

**CAAGGGGCCT**

**>2079.C.Denmark**

**--------------------------------TATACCACGGTTCACACCGTGCAGTCTC**

**CAAGGGGCCT**

**>2079.C.Denmark**

**--------------------------------TATACCACGGTTCACACCGTGCAGTCTC**

**CAAGGGGCCT**

**>2079.C.Denmark**

**--------------------------------TATACCACGGTTCACACCGTGCAGTCTC**

**CAAGGGGCCT**

**>2079.C.Denmark**

**--------------------------------TATACCACGGTTCACACCGTGCAGTCTC**

**CAAGGGGCCT**

**>2079.C.Denmark**

**--------------------------------TATACCACGGTTCACACCGTGCAGTCTC**

**CAAGGGGCCT**

**>**

**>18606A.10.14**

**CCGCTCCAGATGCTGAGATGCCTGTCCCAGACTATACCACGGTTCACACCGTGCAGTCTC**

**CAAGGGGCCT**

**>18606A.10.15**

**CCGCTCCAGATGCTGAGATGCCTGTCCCAGACTATACCACGGTTCACACCGTGCAGTCTC**

**CAAGGGGCCT**

**>18606A.10.16**

**CCGCTCCAGATGCTGAGATGCCTGTCCCAGACTATACCACGGTTCACACCGTGCAGTCTC**

**CAAGGGGCCT**

**>18606B.11.10**

**CCGCTCCAGATGCTGAGATGCCTGTCCCAGACTATACCACGGTTCACACCGTGCAGTCTC**

**CAAGGGGCCT**

**>18606B.11.11**

**CCGCTCCAGATGCTGAGATGCTTGTTCCAGACTATACCACGGTTCACACCGTGCAGTCTC**

**CAAGGGGCCT**

**>18606B.11.12**

**CCGCTCCAGATGCTGAGATGCCTGTCCCAGACTATACCACGGTTCACACCGTGCAGTCTC**

**CAAGGGGCCT**

**>**

**>18607A.1.1**

**CCGCTCCAGATGCTGAGATGCCTGTCCCAGACTATACCACGGTTCACACCGTGCAGTCTC**

**CAAGGGGCCT**

**>18607B.4.4**

**CCGCTCCAGATGCTGAGATGCCTGTCCCAGACTATACCACGGTTCACACCGTGCAGTCTC**

**CAAGGGGCCT**

**>18607B.4.5**

**CCGCTCCAGATGCTGAGATGCCTGTCCCAGACTATACCACGGTTCACACCGTGCAGTCTC**

**CAAGGGGCCT**

**>18607C.2.1**

**CCGCTCCAGATGCTGAGATGCCTGTCCCAGACTATACCACGGTTCACACCGTGCAGTCTC**

**CAAGGGGCCT**

**>18607C.2.2**

**CCGCTCCAGATGCTGAGATGCCTGTCCCAGACTATACCACGGTTCACACCGTGCAGTCTC**

**CAAGGGGCCT**

**>18607C.2.3**

**CCGCTCCAGATGCTGAGATGCCTGTCCCAGACTATACCACGGTTCACACCGTGCAGTCTC**

**CAAGGGGCCT**

**>18607D.2.1A**

**CCGCTCCAGATGCTGAGATGCCTGTCCCAGACTATACCACGGTTCACACCGTGCAGTCTC**

**CAAGGGGCCT**

**>18607D.2.2A**

**CCACTCCAGATGCTGAGATGCCTGTCCCAGACTATACCACGGTTCACACCGTGCAGTCTC**

**CAAGGGGCCT**

**>**

**>2074A.7.10**

**CCGCTCCAGATGCTGAGATGCCTGTCCCAGACTATACCACGGTTCACACCGTGCAGTCTC**

**CAAGGGGCCT**

**>2074A.7.11**

**CCGCTCCAGATGCTGAGATGCCTGTCCCAGACTATACCACGGTTCACACCGTGCAGTCTC**

**CAAGGGGCCT**

**>2074A.7.12**

**CCGCTCCAGATGCTGAGATGCCTGTCCCAGACTATACCACGGTTCACACCGTGCAGTCTC**

**CAAGGGGCCT**

**>2074B.8.13**

**CCGCTCCAGATGCTGAGATGCCTGTCCCAGACTATACCACGGTTCACACCGTGCAGTCTC**

**CAAGGGGCCT**

**>2074B.8.14**

**CCGCTCCAGATGCTGAGATGCCTGTCCCAGACTATACCACGGTTCACACCGTGCAGTCTC**

**CAAGGGGCCT**

**>2074B.8.15**

**CCGCTCCAGATGCTGAGATGCCTGTCCCAGACTATACCACGGTTCACACCGTGCAGTCTC**

**CAAGGGGCCT**

**>**

**>2075A.1.1**

**CCGTTCCAGATGCTGAGATGCCTGTCCCAGACTATACCACGGTTCACACCGTGCAGTCTC**

**CAAGGGGCCT**

**>2075B.2.1**

**CCGCTCCAGATGCTGAGATGCCTGTCCCAGACTATACCACGGTTCACACCGTGCAGTCTC**

**CAAGGGGCCT**

**>2075B.2.2**

**CCGCTCCAGATGCTGAGATGCCTGTCCCAGACTATACCACGGTTCACACCGTGCAGTCTC**

**CAAGGGGCCT**

**>2075C.1.8**

**CCGCTCCAGATGCTGAGATGCCTGTCCCAGACTATACCACGGTTCACACCGTGCAGTCTC**

**CAAGGGGCCT**

**>2075C.1.7**

**CCGCTCCAGATGCTGAGATGCCTGTCCCAGACTATACCACGGTTCACACCGTGCAGTCTC**

**CAAGGGGCCT**

**>**

**>1888.7.9.5.A.Denmark**

**--------------------------------TACACCACGGTTCACACTGTGCAGTCTT**

**CAAGGGGCCT**

**>1888.7.9.5.A.Denmark**

**--------------------------------TACACCACGGTTCACACCGTGCAGTCTC**

**CAAGGGGCCT**

**>1888.7.9.5.A.Denmark**

**--------------------------------TACACCACGGTTCACACCGTGCAGTCTC**

**CAAGGGGCCT**

**>1888.7.9.5.A.Denmark**

**--------------------------------TACACCACGGTTCACACCGTGCAGTCTC**

**CAAGGGGCCT**

**>1888.7.9.5.A.Denmark**

**--------------------------------TACACCACGGTTCACACCGTGCAGTCTC**

**CAAGGGGCCT**

**>1888.7.9.5.A.Denmark**

**--------------------------------TACACCACGGTTCACACCGTGCAGTCTC**

**CAAGGGGCCT**

**>1888.7.9.5.A.Denmark**

**--------------------------------TACACCACGGTTCACACCGTGCAGTCTC**

**CAAGGGGCCT**

**>1888.7.9.5.A.Denmark**

**--------------------------------TACACCACGGTTCACACCGTGCAGTCTC**

**CAAGGGGCCT**

**>1888.7.9.5.A.Denmark**

**--------------------------------TACACCACGGTTCACACCGTGCAGTCTC**

**CAAGGGGCCT**

**>1888.7.9.5.A.Denmark**

**--------------------------------TACACCACGGTTCACACCGTGCAGTCTC**

**CAAGGGGCCT**

**>1888.7.9.5.A.Denmark**

**--------------------------------TACACCACGGTTCACACCGTGCAGTCTC**

**CAAGGGGCCT**

**>1888.7.9.5.A.Denmark**

**--------------------------------TACACCACGGTTCACACCGTGCAGTCTC**

**CAAGGGGCCT**

**>1888.7.9.5.A.Denmark**

**--------------------------------TACACCACGGTTCACACCGTGCAGTCTC**

**CAAGGGGCCT**

**>1888.7.9.5.A.Denmark**

**--------------------------------TACACCACGGTTCACACCGTGCAGTCTC**

**CAAGGGGCCT**

**>1888.7.9.5.A.Denmark**

**--------------------------------TACACCACGGTTCACACCGTGCAGTCTC**

**CAAGGGGCCT**

**>1888.7.9.5.A.Denmark**

**--------------------------------TACACCACGGTTCACACCGTGCAGTCTC**

**CAAGGGGCCT**

**>**

**>1899.8.6.28.A.Denmark**

**--------------------------------TACACCACGGTTCACACCGTGCAGTCTC**

**CAAGGGGCCT**

**>1899.8.6.28.A.Denmark**

**--------------------------------TACACCACGGTTCACACCGTGCAGTCTC**

**CAAGGGGCCT**

**>1899.8.6.28.A.Denmark**

**--------------------------------TACACCACGGTTCACACCGTGCAGTCTC**

**CAAGGGGCCT**

**>1899.8.6.28.A.Denmark**

**--------------------------------TACACCACGGTTCACACCGTGCAGTCTC**

**CAAGGGGCCT**

**>1899.8.6.28.A.Denmark**

**--------------------------------TACACCACGGTTCACACCGTGCAGTCTC**

**CAAGGGGCCT**

**>1899.8.6.28.A.Denmark**

**--------------------------------TACACCACGGTTCACACCGTGCAGTCTC**

**CAAGGGGCCT**

**>1899.8.6.28.A.Denmark**

**--------------------------------TACACCACGGTTCACACCGTGCAGTCTC**

**CAAGGGGCCT**

**>1899.8.6.28.A.Denmark**

**--------------------------------TACACCACGGTTCACACCGTGCAGTCTC**

**CAAGGGGCCT**

**>1899.8.6.28.A.Denmark**

**--------------------------------TACACCACGGTTCACACCGTGCAGTCTC**

**CAAGGGGCCT**

**>1899.8.6.28.A.Denmark**

**--------------------------------TACACCACGGTTCACACCGTGCAGTCTC**

**CAAGGGGCCT**

**>1899.8.6.28.A.Denmark**

**--------------------------------TACACCACGGTTCACACCGTGCAGTCTC**

**CAAGGGGCCT**

**>1899.8.6.28.A.Denmark**

**--------------------------------TACACCACGGTTCACACCGTGCAGTCTC**

**CAAGGGGCCT**

**>**

**>1899.8.6.29.A.Denmark**

**--------------------------------TACACCACGGTTCACACCGTGCAGTCTC**

**CAAGGGGCCT**

**>1899.8.6.29.A.Denmark**

**--------------------------------TACACCACGGTTCACACCGTGCAGTCTC**

**CAAGGGGCCT**

**>1899.8.6.29.A.Denmark**

**--------------------------------TACACCACGGTTCACACCGTGCAGTCTC**

**CAAGGGGCCT**

**>1899.8.6.29.A.Denmark**

**--------------------------------TACACCACGGTTCACACCGTGCAGTCTC**

**CAAGGGGCCT**

**>1899.8.6.29.A.Denmark**

**--------------------------------TACACCACGGTTCACACCGTGCAGTCTC**

**CAAGGGGCCT**

**>1899.8.6.29.A.Denmark**

**--------------------------------TACACCACGGTTCACACCGTGCAGTCTC**

**CAAGGGGCCT**

**>1899.8.6.29.A.Denmark**

**--------------------------------TACACCACGGTTCACACCGTGCAGTCTC**

**CAAGGGGCCT**

**>1899.8.6.29.A.Denmark**

**--------------------------------TACACCACGGTTCACACCGTGCAGTCTC**

**CAAGGGGCCT**

**>1899.8.6.29.A.Denmark**

**--------------------------------TACACCACGGTTCACACCGTGCAGTCTC**

**CAAGGGGCCT**

**>1899.8.6.29.A.Denmark**

**--------------------------------TACACCACGGTTCACACCGTGCAGTCTC**

**CAAGGGGCCT**

**>1899.8.6.29.A.Denmark**

**--------------------------------TACACCACGGTTCACACCGTGCAGTCTC**

**CAAGGGGCCT**

**>1899.8.6.29.A.Denmark**

**--------------------------------TACACCACGGTTCACACCGTGCAGTCTC**

**CAAGGGGCCT**

**>1899.8.6.29.A.Denmark**

**--------------------------------TACACCACGGTTCACACCGTGCAGTCTC**

**CAAGGGGCCT**

**>1899.8.6.29.A.Denmark**

**--------------------------------TACACCACGGTTCACACCGTGCAGTCTC**

**CAAGGGGCCT**

**>**

**>TRYP A**

**>18846A.1.1**

**CGGATTCTTTCCGGCGTCTTTTGA**

**>18846A.1.4**

**CGGATTCTTTCCGGCGTCTTTTGA**

**>18846B.2.5**

**CGGATTCTTTCCGGCGTCTTTTGA**

**>18846B.2.6**

**CGGATTCTTTCCGGCGTCTTTTGA**

**>18846C.G1.Denmark**

**CGGATTCTTTCCGGCGTCTTTTGA**

**>18846C.G2.Denmark**

**CGGATTCTTTCCGGCGTCTTTTGA**

**>**

**>18607A.1.1**

**CGGATTCTTTCCGGCGTCTTTTGA**

**>18607A.1.2**

**CGGATTCTTTCCGGCGTCTTTTGA**

**>18607A.1.3**

**CGGATTCTTTCCGGCGTCTTTTGA**

**>18607B.2.4**

**CGGATTCTTTCCGGCGTCTTTTGA**

**>18607B.2.5**

**CGGATTCTTTCCGGCGTCTTTTGA**

**>18607C.G1.Denmark**

**CGGATTCTTTCCGGCGTCTTTTGA**

**>18607C.G2.Denmark**

**CGGATTCTTTCCGGCGTCTTTTGA**

**>**

**>2079A.U4**

**CGGATTCTTTCCGGCGTCTTTTGA**

**>2079A.U11**

**CGGATTCTTTCCGGCGTCTTTTGA**

**>2079B.1.1**

**CGGATTCTTTCCGGCGTCTTTTGA**

**>2079B.1.12**

**CGGATTCTTTCCGGTGTCTTTTGA**

**>2079C.G1.Denmark**

**CGGATTCTTTCCGGCGTCTTTTGA**

**>2079C.G2.Denmark**

**CGGATTCTTTCCGGCGTCTTTTGA**

**>2079C.G3.Denmark**

**CGGATTCTTTCCGGCGTCTTTTGA**

**>**

**>2075A.11**

**CGGA---TTTCCGGCGTCTTTTGA**

**>2075A.12**

**CGGA---TTTCCGGCGTCTTTTGA**

**>2075A.13**

**CGGA---TTTCCGGCGTCTTTTGA**

**>2075A.14**

**CGGA---TTTCCGGCGTCTTTTGA**

**>2075A.15**

**CGGA---TTTCCGGCGTCTTTTGA**

**>**

**>TRYP B**

**>18846A.2**

**TTTTTGGTCCTCGCAAGAGGTCCTTTTACGGGAA**

**>18846A.3**

**TTTTTGGTCCTCGCAAGAGGTCCTTTTACGGGAA**

**>18846A.4**

**TTTTTGGTCCTCGCAAGAGGTCCTTTTACGGGAA**

**>18846A.5**

**TTTTTGGTCCTCGCAAGAGGTCCTTTTACGGGAA**

**>18846A.6**

**TTTTTGGTCCTCGCAAGAGGTCCTTTTACGGGAA**

**>18846A.7**

**TTTTTGGTCCTCGCAAGAGGTCCTTTTACGGGAA**

**>18846A.8**

**TTTTTGGTCCTCGCAAGAGGTCCTTTTACGGGAA**

**>18846B.G1.Denmark**

**TTTTTGGTCCTCGCAAGAGGTCCTTTTACGGGAA**

**>**

**>18607.T4.4**

**TTTTTGGTCCTCGCAAGAGGTCCTTTTACGGGAA**

**>18607A.4F**

**TTTTTGGTCCTCGCAAGAGGTCCTTTTACGGGAA**

**>18607A.5F**

**TTTTTGGTCCTCGCAAGAGGTCCTTTTACGGGAA**

**>18607B.A**

**TTTTTGGTCCTCGCAAGAGGTCCTTTTACGGGAA**

**>18607B.C**

**TTTTTGGTCCTCGCAAGAGGTCCTTTTACGGGAA**

**>18607B.E**

**TTTTTGGTCCTCGCAAGAGGTCCTTTTACGGGAA**

**>18607B.F**

**TTTTTGGTCCTCGCAAGAGGTCCTTTTACGGGAA**

**>18607C.G1.Denmark**

**TTTTTGGTCCTCGCAAGAGGTCCTTTTACGGGAA**

**>18607C.G2.Denmark**

**TTTTTGGTCCTCGCAAGAGGTCCTTTTACGGGAA**

**>18607C.G3.Denmark**

**TTTTTGGTCCTCGCAAGAGGTCCTTTTACGGGAA**

**>**

**>2079A.2.1**

**TTTTTGGTCCTCGCAAGAGGTCCTTTTACGGGAA**

**>2079A.2.3**

**TTTTTGGTCCTCGCAAGAGGTCCTTTTACGGGAA**

**>2079B.7.1**

**TTTTTGGTCCTCGCAAGAGGTCCTTTTACGGGAA**

**>2079B.7.2**

**TTTTTGGTCCTCGCAAGAGGTCCTTTTACGGGAA**

**>2079B.7.3**

**TTTTTGGTCCTCGCAAGAGGTCCTTTTACGGGAA**

**>2079B.7.4**

**TTTTTGGTCCTCGCAAGAGGTCCTTTTACGGGAA**

**>2079B.7.5**

**TTTTTGGTCCTCGCAAGAGGTCCTTTTACGGGAA**

**>**

**>2077A.1.1**

**TTTTTGGTCCTCGCAAGAGGTCCTTTTACGGGAA**

**>2077A.1.2**

**TTTTTGGTCCTCGCAAGAGGTCCTTTTACGGGAA**

**>2077A.1.3**

**TTTTTGGTCCTCGCAAGAGGTCCTTTTACGGGAA**

**>2077A.1.4**

**TTTTTGGTCCTCGCAAGAGGTCCTTTTACGGGAA**

**>**

**>2074A.1**

**TTTTTGGTCCTCGCAAGAGGTCCTTTTACGGGAA**

**>2074A.2**

**TTTTTGGTCCTCGCAAGAGGTCCTTTTACGGGAA**

**>2074A.3**

**TTTTTGGTCCTCGCAAGAGGTCCTTTTACGGGAA**
